# Calibration of local chemical pressure by optical probe

**DOI:** 10.1093/nsr/nwad190

**Published:** 2023-07-10

**Authors:** Xiao Zhou, Mei-Huan Zhao, Shan-Ming Yao, Hongliang Dong, Yonggang Wang, Bin Chen, Xianran Xing, Man-Rong Li

**Affiliations:** Key Laboratory of Bioinorganic and Synthetic Chemistry of Ministry of Education, School of Chemistry, Sun Yat-sen University, Guangzhou 510275, China; Key Laboratory of Bioinorganic and Synthetic Chemistry of Ministry of Education, School of Chemistry, Sun Yat-sen University, Guangzhou 510275, China; Key Laboratory of Bioinorganic and Synthetic Chemistry of Ministry of Education, School of Chemistry, Sun Yat-sen University, Guangzhou 510275, China; Center for High Pressure Science and Technology Advanced Research, Shanghai 201203, China; School of Materials Science and Engineering, Peking University, Beijing 100871, China; Center for High Pressure Science and Technology Advanced Research, Shanghai 201203, China; Beijing Advanced Innovation Center for Materials Genome Engineering, Institute of Solid State Chemistry, University of Science and Technology Beijing, Beijing 100083, China; Key Laboratory of Bioinorganic and Synthetic Chemistry of Ministry of Education, School of Chemistry, Sun Yat-sen University, Guangzhou 510275, China; School of Science, Hainan University, Haikou 570228, China

**Keywords:** chemical pressure, high pressure, ion substitution, optical parameter, spectra shift, pressure calibration

## Abstract

Chemical stabilization of a high-pressure metastable state is a major challenge for the development of advanced materials. Although chemical pressure (*P*_chem_) can effectively simulate the effect of physical pressure (*P*_phy_), experimental calibration of the pressure passed to local structural motifs, denoted as local chemical pressure (*P*_chem-_*_Δ_*) which significantly governs the function of solid materials, remains absent due to the challenge of probing techniques. Here we establish an innovative methodology to experimentally calibrate the *P*_chem-_*_Δ_* and build a bridge between *P*_chem_ and *P*_phy_ via an optical probe strategy. Site-selective Bi^3+^-traced *RE*VO_4_ (*RE* = Y, Gd) is adopted as a prototype to introduce Bi^3+^ optical probes and on-site sense of the *P*_chem-_*_Δ_* experienced by the *RE*O_8_ motif. The cell compression of *RE*_0.98_Bi_0.02_VO_4_ under *P*_phy_ is chemically simulated by smaller-ion substitution (Sc^3+^ → *RE*^3+^) in *RE*_0.98-_*_x_*Sc*_x_*Bi_0.02_VO_4_. The consistent red shift (*Δλ*) of the emission spectra of Bi^3+^, which is dominated by locally pressure-induced *RE*O_8_ dodecahedral variation in *RE*_0.98_Bi_0.02_VO_4_ (*P*_phy_) and *RE*_0.98-_*_x_*Sc*_x_*Bi_0.02_VO_4_ (*P*_chem-_*_Δ_*), respectively, is evidence of their similar pressure-dependent local structure evolution. This innovative *Δλ*-based experimental calibration of *P*_chem-_*_Δ_* in the crystal-field dimension portrays the anisotropic transmission of *P*_chem_ to the local structure and builds a bridge between *P*_chem-_*_Δ_* and *P*_phy_ to guide a new perspective for affordable and practical interception of metastable states.

## Teaser

Pressure-dependent spectral evolution sensed by onsite optical probe builds a conceivable bridge between chemical and physical pressures, and methodologically develops an experimental approach to scale local chemical pressure.

## INTRODUCTION

The application of high pressure adds an additional dimension to chemical and thermodynamic space, effectively alters the structural, chemical, and physical features of materials, boosts discoveries of novel states of matter [1–5], such as high-*T*_C_ superconductivity [6–8], unprecedented compounds [9], unexpected stoichiometries [10], superhard materials [11], and exotic quantum magnetoelectric behaviors [12–14]. However, the high-pressure state of materials is metastable, and the emergent phases and phenomena usually disappear after decompression [15]. Although some metastable polymorphs prepared via high-pressure and high-temperature approaches can be ‘frozen’ to ambient pressure by temperature quenching before decompressing, the high cost and poor yield (mostly 10^1^–10^2^ mg per batch) drastically reduce their practical applications [16–19]. Herein, scaled-up capturing of the metastable phases at ambient pressure is highly anticipated. Physical (mechanical)-pressure (*P*_phy_) compresses the unit-cell dimension, while the equivalent effect can also be mimicked by chemical approaches (chemical pressure, *P*_chem_) [20], such as interfacial compressive strain (external *P*_chem_), size-dependent ionic substitution (internal *P*_chem_) and stabilization of the bulky phase [21–23]. External *P*_chem_ has been successfully applied to stabilize the metastable phases in hetero-junction thin-film forms at ambient pressure [24–29], where the compressive/tensile stress established by lattice mismatch between substrate and epitaxial heterojunction thin-film has been estimated to reach several positive/negative gigapascal (GPa). Internal *P*_chem_ in a given compound can be generated by chemical substitution with smaller/larger ions in order to induce cell contraction/expansion (equivalent to positive/negative *P*_chem_) [21,30–35]. For example, the quantum critical temperatures of Fe-based superconductors BaFe_2_As_2-_*_x_*P*_x_* and FeSe_1-_*_x_*S*_x_* can be dramatically increased by partial replacement of As/Se by smaller P/S, respectively, yielding equivalent performance compared to the un-doped bulk analogs under *P*_phy_ [31,35]. The above discoveries show that *P*_chem_ can simulate *P*_phy_ at scaled-up yield and lower cost to a great extent.

Building a ‘bridge’ between *P*_chem_ and *P*_phy_ is of significance in designing novel materials and stabilizing the metastable state. Unlike the experimentally optical [36] and electrical [37] calibration of *P*_phy_, hitherto, the *P*_chem_ has been roughly computed on the crystallographic unit-cell-volume (*V*) scale by the state for solids equation [38]. However, the spatial resolution of this *V*-based estimation limits further in-depth exploration of the *P*_chem_ effect, since the physical properties of solids and the intercepting limitation of metastable state are highly sensitive to the pressure effect of functional motifs on the local environment [39–42]. The closer the *P*_chem_ simulates the evolution of local structure under *P*_phy_, the more likely it is to chemically intercept the metastable state [43]. So, it is essential to weigh up the *P*_chem_ effect on the local structure scale of materials. However, this has not yet been experimentally realized as yet. It is highly desirable to methodologically reveal the relationship between *P*_phy_/- local-*P*_chem_ (*P*_chem-_*_Δ_*) and chemical variates (such as substitution concentration), to mimic the *P*_phy_ effect by using chemical approaches.

**Figure 1.**
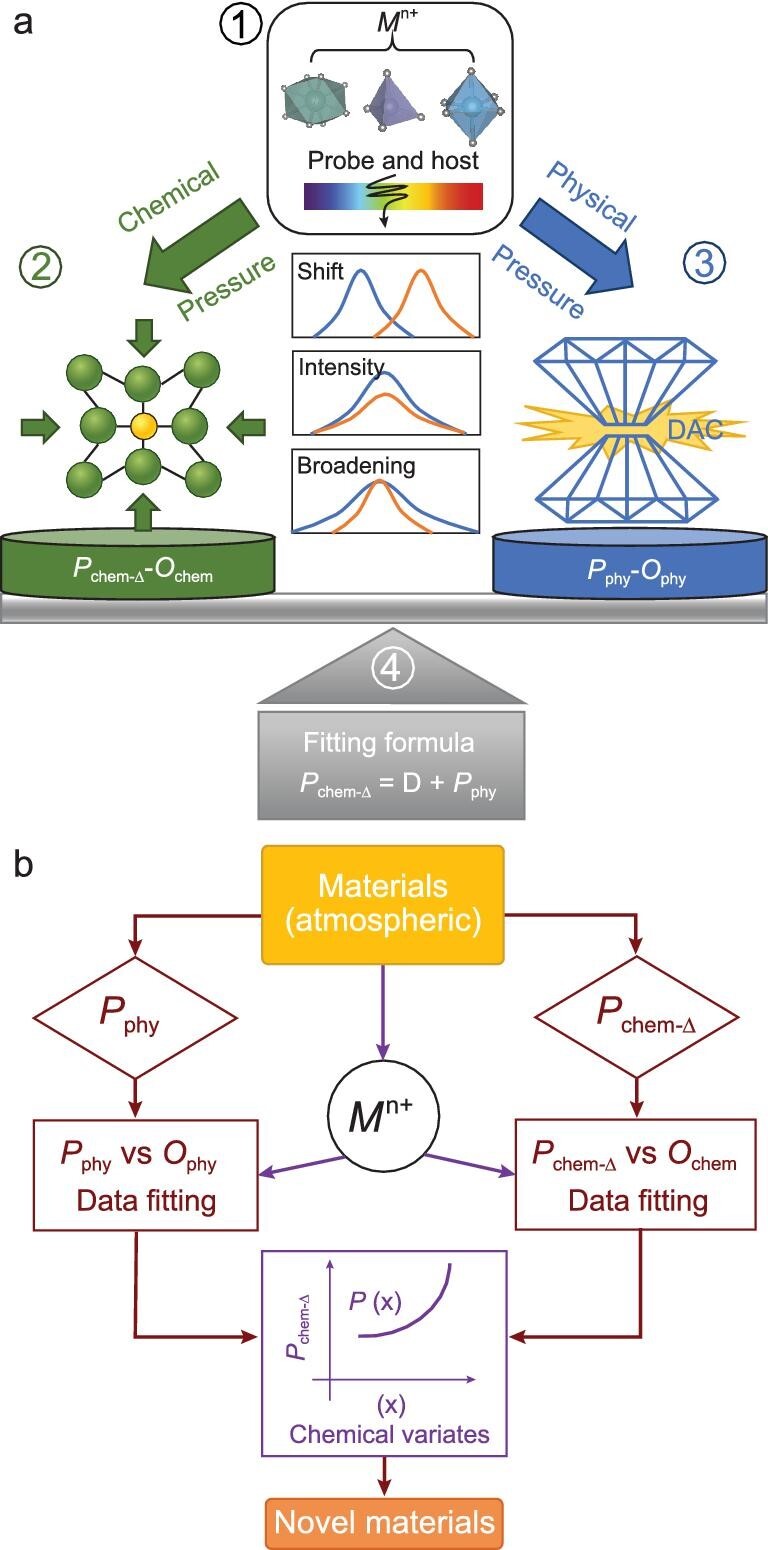
The methodological logic (a) and implementation process diagrams (b) of local chemical pressure (*P*_chem-_*_Δ_*) calibration via the optical parameters (*O*_phy_/*O*_chem_) of the on-site optical probe *M^n^*^+^.

Optical parameters (such as absorption spectra and CIE color coordinates) of site-selective chromophore ions can echo on the local surroundings and thus sensitively probe the alteration of the corresponding crystal field under pressure [44]. Accordingly, the *P*_chem_ delivered to local motifs could be sensed by the evolution of onsite optical parameters. The pressure experienced by local motifs (traced by the site-selective optical probe) is denoted as the local chemical pressure (*P*_chem-_*_Δ_*), manifested by local expansion, contraction, or distortion of the polyhedron beyond *V*-variation. Here, we innovatively developed an optical-probe approach to calibrate the *P*_chem-_*_Δ_*, as schematically illustrated in Fig. 1a. (1) First, the site-selective ion (*M^n^*^+^) incorporated into a specific polyhedron acts as an optical-probe, stemming from which the optical information effectively sounds the pressure-dependent variation in the crystal field. (2) The physically *in-situ* optical parameters (*O*_phy_) evolution in (1) collected in a diamond anvil cell (DAC) reveals the structural and optical evolution under *P*_phy_ [36,45]. Here the *P*_phy_–*O*_phy_ can be fitted by the formula that conforms to a trend or theory. (3) Parallel substitution of the substance in (1) by smaller ions draws cell contraction so that the impact of *P*_chem-_*_Δ_* on the local environment (crystal field) can be expressed by the optical characteristics of the probe. Thus, the *P*_chem-_*_Δ_* experienced by the crystal field (local structure) can be depicted by the optical parameters (*O*_chem_) at different substitution levels. (4) Finally, the correlation of *O*_phy_ and *O*_chem_ quantitatively calibrates the *P*_chem-_*_Δ_* as shown in the schematic diagram in Fig. 1b.

In this work, we take *RE*VO_4_ (*RE* = Gd, Y) as the host, and incorporate Bi^3+^ ion as the luminescence center into the *RE*O_8_ site to form a BiO_8_ dodecahedron. The shift of the Bi^3+^ emission peak (*Δλ*) is adopted as the optical parameter, to paradigmatically develop a method for experimental calibration of *P*_chem-_*_Δ_*. The *P*_phy_-dependent optical spectra (*Δλ*_phy_) of the selected *RE*_0.98_Bi_0.02_VO_4_ was measured in a DAC to extract the *P*_phy_–*Δλ*_phy_ information below 8.5 and 7.0 GPa, the phase-transition pressure points for Y_0.98_Bi_0.02_VO_4_ and Gd_0.98_Bi_0.02_VO_4_, respectively [46,47]. In contrast, the *P*_chem_/*P*_chem-_*_Δ_* was induced by substitution of Gd^3+^/Y^3+^ with isovalent but smaller Sc^3+^ in *RE*_1-_*_x-y_*Sc*_x_*Bi*_y_*VO_4_, where the *P*_chem_ distributed on local (*RE*_1−_*_x_*_−_*_y_*Sc*_x_*Bi*_y_*)O_8_ was depicted by *P*_chem-_*_Δ_* and the related optical parameter (*Δλ*_chem_). Accordingly, the *Δλ*-mirrored structural evolution builds a scale bar between *P*_phy_ and *P*_chem-_*_Δ_*, and thus realizes experimental calibration of *P*_chem-_*_Δ_*, which is important in exploring the limitation of chemical stabilization of metastable states.

## RESULTS AND DISCUSSION

### Picturing of optical-probe traced *RE*VO_4_

*RE*VO_4_ (*RE* = Y, Gd) is chosen as a paradigm to bridge the *P*_phy_ and *P*_chem_ (*V*-based)-*P*_chem-_*_Δ_* (local-structure based) in this work. YVO_4_ crystallizes in a zircon-type tetragonal structure (Fig. S1 top, *I*4_1_/*amd*, No. 141), where Y^3+^ and V^5+^ ions are in edge-shared YO_8_ dodecahedral and isolated VO_4_ tetrahedral coordination, respectively. Usually, the VO_4_ tetrahedron is regarded as a relatively rigid unit [48]. The YO_8_ dodecahedron consists of four longer and four shorter bonds, denoted as Y-O1 and Y-O2, respectively (Fig. S1 bottom). To further validate the accuracy and applicability through solitary experiments, the isostructural GdVO_4_ is also adopted for parallel studies [47].

The excited electron configuration of Bi^3+^ ([Xe]4*f*^14^5*d*^10^6*s*^2^) is very sensitive to the crystal field [49–52], so that the evolution of luminescence properties of Bi^3+^ under pressure can witness the change in the crystal field on-site, namely the local structure surrounding Bi^3+^. The ionic radius of Bi^3+^ (1.17 Å at a coordination number (CN) of 8) is highly comparable to those of *RE*^3+^ (Y^3+^/Gd^3+^ = 1.019/1.053 Å at CN = 8) [53]. Thereby, Bi^3+^ is prone to site-selectively substitute the dodecahedral *RE*^3+^ to form a BiO_8_ unit as evidenced by crystallographic analyses (Fig. S2 and Table S1). It should be noted that: (1) the overall crystal structure symmetry of the host should not be modified by the accommodation of an ‘optical probe’, and (2) the optical parameter of the probe should be suitable for unambiguous observation. Accordingly, a 2% Bi^3+^-doping concentration was introduced into the sample as the starting point, namely, *RE*_0.98_Bi_0.02_VO_4_ (*RE* = Y, Gd, Fig. S3), since its emission intensity and range can be well monitored by most common optical instruments.

### Pressure-dependent structural evolution

#### Cell compression under *p*_phy_

**Figure 2.**
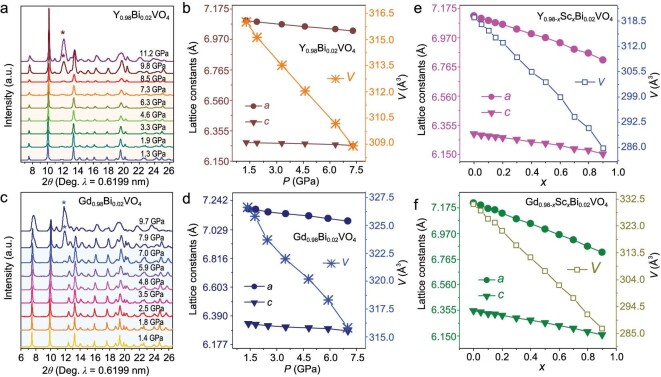
*In situ* pressure-dependent synchrotron radiation patterns of Y_0.98_Bi_0.02_VO_4_ (a) and Gd_0.98_Bi_0.02_VO_4_ (c), and the corresponding lattice parameters (*V, a, c*) variation (b and d). The lattice parameters (*V, a, c*) variation of Y_0.98-_*_x_*Sc*_x_*Bi_0.02_VO_4_ (e) and Gd_0.98-_*_x_*Sc*_x_*Bi_0.02_VO_4_ (f) with different ion substitution concentrations *x*.

The *in situ* pressure-dependent synchrotron powder X-ray diffraction (SPXD) runs of Y_0.98_Bi_0.02_VO_4_ were conducted in a DAC with silicone oil as PTM (pressure transmitting media), to generate macroscopically isotropic *P*_phy_. As shown in Fig. 2a, the *I*4_1_/*amd* structure is maintained below ∼8.5 GPa as reported [54]. The *P*_phy_-dependent dimension evolution is extracted by fitting the diffraction patterns collected between 1.3–8.5 GPa (Table S1). The *P*_phy_ versus lattice-parameter plots demonstrate clear cell contraction upon pressing (Fig. 2b). The compression by 0.0683 Å in the *a*-axis at 8.5 GPa is more than that in the *c*-direction (0.0213 Å), demonstrating anisotropic compressibility. Above 8.5 GPa, the anomalous broadening and strengthening scheelite (112) reflection (marked by * in Fig. 2a) signifies an irreversible transformation from zircon (*I*4_1_/*amd*) to scheelite phase (*I*4_1_/*a*). For Gd_0.98_Bi_0.02_VO_4_, similar variation of *a, c* and *V* is observed below 7.0 GPa as shown in Fig. 2d. Above 7.0 GPa, signals of the scheelite phase (marked by * in Fig. 2c) appear. The compressibility by *V* reaches ∼2.2% and 3.3% below 8.5 and 7.0 GPa for Y_0.98_Bi_0.02_VO_4_ and Gd_0.98_Bi_0.02_VO_4_, respectively. Obviously, the cell is more compressible along the *a*-axis than along the *c*-axis, indicating anisotropic lattice contraction in *RE*_0.98_Bi_0.02_VO_4_ under *P*_phy_.

#### Cell contraction under *p*_chem_

**Figure 3.**
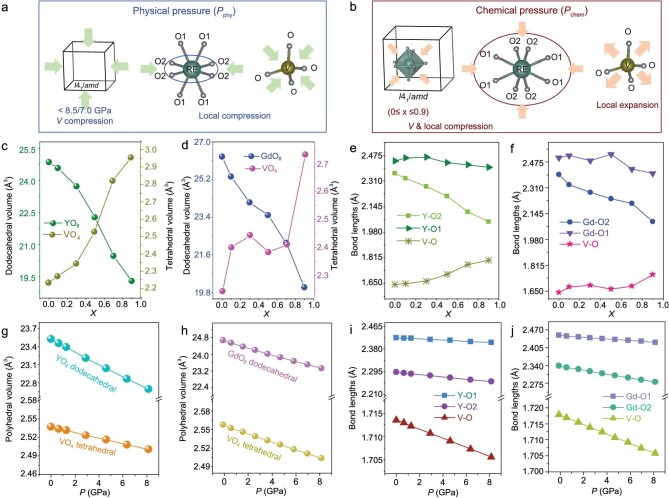
The diagram of structure evolution in the *RE*VO_4_ system under physical pressure (a) and chemical pressure (b). The evolution of the polyhedral volume (dodecahedral *RE*O_8_ and tetrahedral VO_4_) and the bond lengths (*RE*−O, V−O) in Y_0.98-_*_x_*Sc*_x_*Bi_0.02_VO_4_ (c and e) and Gd_0.98-_*_x_*Sc*_x_*Bi_0.02_VO_4_ (d and f) with different ion substitution concentrations *x*, respectively. The evolution (by DFT calculations) of the polyhedral volume (dodecahedral *RE*O_8_ and tetrahedral VO_4_) and the bond lengths (*RE*−O, V−O) in Y_0.98_Bi_0.02_VO_4_ (g and i) and Gd_0.98_Bi_0.02_VO_4_ (h and j) under high pressure, respectively.

*P*_chem_ is an internal force caused by lattice strain with chemical modifications [20]. Isovalent ionic substitution by smaller ions with similar external electronic configuration is expected to generate positive *P*_chem_ in the *V*-dimension [38,55]. In this work, substituting Sc^3+^ ([Ar]3*s*^2^3*p*^6^) for *RE*^3+^ in *RE*_0.98_Bi_0.02_VO_4_ (*RE* = Y^3+^ [Kr]4*s*^2^3*d*^10^4*p*^6^, Gd^3+^ [Xe]4*f*^7^) is applied to exert *P*_chem_, in that, (1) Sc^3+^ has a small ionic radius (0.87 Å at CN = 8, about 0.15 and 0.18 Å smaller than that of Y^3+^ and Gd^3+^, respectively); (2) ScVO_4_ has been reported to adopt the isostructural tetragonal *I*4_1_/*amd* polymorph, thus, substituting Sc for Y/Gd is desired to form an infinite solid solution, and result in a volumetric contraction up to 11.2% and 14.3% in YVO_4_ and GdVO_4_, respectively [54,56]. The PXD patterns of Y_0.98-_*_x_*Sc*_x_*Bi_0.02_VO_4_ and Gd_0.98-_*_x_*Sc*_x_*Bi_0.02_VO_4_ (0 ≤ *x* ≤ 0.9) show the pure tetragonal (*I*4_1_/*amd*) phase for the whole *RE*_0.98-_*_x_*Sc*_x_*Bi_0.02_VO_4_ series (Figs S4 and S5). The diffraction peaks systematically shift toward a higher angle with incremental *x* as highlighted (Fig. S4), suggesting continuous cell contraction. The lattice constants refined from PXD data show monotonic cell compression with *a, c*, and *V* in an approximately linear way with increasing substitution levels of Sc as shown in Fig. 2e and f, satisfying Vegard's law in a continuous solid solution and manifesting positive *P*_chem_. The *a*/*c* axes decrease by 4.3%/2.2% and 5.5%/3.0% for Y_0.98-_*_x_*Sc*_x_*Bi_0.02_VO_4_ and Gd_0.98-_*_x_*Sc*_x_*Bi_0.02_VO_4_, respectively. Here the contraction is slightly less pronounced along the *c*-direction than that along the *a*-direction, suggesting a slightly anisotropic *P*_chem_ effect, which is consistent with the performance observed under *P*_phy_ (Figs 2 and S4). Above certain *P*_phy_ (8.5/7.0 GPa), the transition from zircon to scheelite polymorph arises. In contrast, the effect of *P*_chem_ is intrinsic, the substitution of Sc^3+^ significantly compresses the (*RE*/Bi^3+^)O_8_ dodecahedron, which eventually drives smaller *V* (compressibility of ∼11.2% and 14.3% for Y- and Gd-analogs) without any phase transition (Fig. 3b) compared to the *P*_phy_ effect in Fig. 2a and c, where phase transition occurs at compressibility beyond ∼2.2% and 3.3% for *RE* = Y and Gd, respectively. This discrepancy of phase evolution under *P*_phy_ and *P*_chem_ suggests that a higher substitution level may result in a larger deviation referenced to the dimension of ScVO_4_.

In Fig. 3a and b, the diagrams display the contraction of the cell volume *V* under different pressures. While *P*_chem_ occurs from within the lattice (Fig. 3a), *P*_phy_ is delivered from the outside in. Varied evolution of local structures under *P*_phy_ and *P*_chem_ is caused by different pressure transfer properties that do not have an obvious effect on *V*.

#### Local structure evolution

The *P*_phy_-dependent Raman spectra of *RE*_0.98_Bi_0.02_VO_4_ further verify the above conclusions (Fig. S6). The Raman active modes of Y_0.98_Bi_0.02_VO_4_ are similar to those reported by Manjón *et al.* [57]. The three Raman peaks marked by asterisks at ∼230 (external modes), ∼376, and ∼867 cm^−1^ (internal modes), which are attributed to the scheelite-type YVO_4_, gradually emerge above 8.6 GPa (Fig. S6). The noticeable shift of Raman peaks can be observed in the Raman spectra from 0.6 to 7.9 GPa in the zircon-type tetragonal zone. These Raman peaks originate from the internal (∼384, ∼491, ∼819, ∼842, ∼893 cm^−1^, internal motion of VO_4_) and external (∼160, ∼258 cm^−1^, transitions and rotations of VO_4_) modes as a whole with respect to Y^3+^ [46]. As *P*_phy_ increases, the Raman peaks of internal modes, especially the one at ∼893 cm^−1^, shift to a higher frequency, indicating V−O bond strengthening due to a charge transfer rather than bond lengthening [46]. On the contrary, the external models are rarely sensitive to pressure, and even slightly evolve toward the low frequency (∼258 cm^−1^) side, which is consistent with previous reports [57]. Thereby, the translations and rotations of VO_4_ tetrahedral as a whole have lower sensitivity to pressure compared to the Y−O group. Detailed pressure-dependent variation of the interatomic distances in YVO_4_ can be referenced to the literature [54]. In contrast, the Y−O2 bond shortens significantly along the *a*-axis in the pressurized Y_0.98_Bi_0.02_VO_4_, and adds a major contribution to volumetric contraction, whereas the variation of Y−O1 and V−O is less distinct as seen from the Raman spectra (Fig. S6a). The *P*_phy_-dependent local structural evolution in Gd_0.98_Bi_0.02_VO_4_ is consistent with what was observed in the parent Y-analog (Fig. S6b).

The local structure variation evidencing *P*_chem_ in *RE*_0.98-_*_x_*Sc*_x_*Bi_0.02_VO_4_ was revealed by crystallographic analysis (Fig. S2; Tables S1 and S2). As shown in Fig. 3c and d, the spatial distribution of local dodecahedral *RE*O_8_ and tetrahedral VO_4_ vary oppositely under chemical pressure. With increasing substitution concentration, the volume of the local *RE*O_8_ dodecahedron in *RE*_0.98_*_x_*Sc*_x_*Bi_0.02_VO_4_ undergoes contraction, while that of the local tetrahedral VO_4_ is expanded (Fig. 3c and d). Although the *x*-dependent local volume of VO_4_ somewhat fluctuates (Fig. 3d) in Gd_0.98-_*_x_*Sc*_x_*Bi_0.02_VO_4_, the overall trend is similar to that of the Y-analog. The *x*-dependent variation of bond lengths in Fig. 3e (Y/V−O) and f (Gd/V−O) imply more anisotropic local structure evolution. When Sc^3+^ is site-selectively introduced into dodecahedral *RE*O_8_, bond compression (especially the Y−O2 and Gd−O2) arises, passing the ‘pressure’ to the local surroundings (*RE*O_8_, VO_4_). The bond-length variation trend of Y/Gd−O2 is remarkably parallel to its sensitivity to *P*_phy_ and *P*_chem_. In contrast, the local VO_4_ motifs in *RE*_0.98-_*_x_*Sc*_x_*Bi_0.02_VO_4_ (Fig. 3e and f) are expanded, attributed to the simultaneous stretching of the oxygen-sharing Y−O2 bond and the edge-sharing Y-O1 bond under *P*_chem_ [58].

The local structure evolutions of *RE*VO_4_ under *P*_phy_ were simulated by density functional theory (DFT) calculations, since the *in situ* high-pressure SPXD data are insufficient for decent structural refinements, because of a bunch of overexposure spots as shown in Supplementary Fig. S7. Both polyhedral volumes and bond lengths of *RE*_0.98-_*_x_*Sc*_x_*Bi_0.02_VO_4_ decrease with increasing *P*_phy_ (Fig. 3g–j). The compression trend of *RE*O_8_ under *P*_phy_ is similar to that of *RE*_0.98-_*_x_*Sc*_x_*Bi_0.02_O_8_ under *P*_chem_, which explains the similarities in optical evolutions under *P*_phy_ and *P*_chem_ as described in the next part of this article. The contraction of VO_4_ under physical pressure results from overall external compression, in contrast, the expansion of VO_4_ under *P*_chem_ stems from the stretching of oxygen shared with the contracted *RE*_0.98-_*_x_*Sc*_x_*Bi_0.02_VO_8_ motifs. However, the opposite trends of VO_4_ under *P*_phy_ and *P*_chem_ do not affect the optical properties in an obvious way.

The diagrams depict the local structure evolution under *P*_phy_ (Fig. 3a) and *P*_chem_ (Fig. 3b). The local compression of *RE*O_8_ in *RE*_0.98_Bi_0.02_VO_4_ under *P*_phy_ is more obvious along the *RE*−O2 bond, according to reports in the literature and theoretical calculations, whereas the compression of the tetrahedron is not obvious because of its stable rigid structure (Fig. 3a). The local motifs (*RE*O_8_, VO_4_) of *RE*_0.98-_*_x_*Sc*_x_*Bi_0.02_VO_4_ are shown in Fig. 3b. *RE*O_8_ is compressed as *x* increases, but VO_4_ is expanded. The bond length values are referred to in Table S2. In other words, *RE*O_8_ and VO_4_ locally ‘feel’ positive and negative pressure, respectively, giving an overall positive pressure in the *V*-scale. The chemical modification approach effectively simulates the evolution of volume (*V*) and the concerned group (*RE*O_8_) under *P*_phy_, which is the basis of the feasibility of our methodology.

### ***Δλ***-based chemical pressure calibration

Crystal structure changes, such as cell contraction, atomic-distance shortening, structure distortion, and others, are relatively pure/clean and integral in atomic scales under *P*_phy_ with PTM. So far, it still lacks an experimental assessment of *P*_chem-_*_Δ_*, which is critical to the properties of solid-state materials [58–60]. Optical parameters are the highly detectable media for sensing changes in the crystal field determined by the local environment. Here, site-selective luminescence ion (Bi^3+^) as an optical probe is applied to sense the crystal-field scaled *P*_chem-_*_Δ_*, that is, the lattice internal force distributed on the local group.

#### Luminescence properties of site-selective Bi^3+^

**Figure 4.**
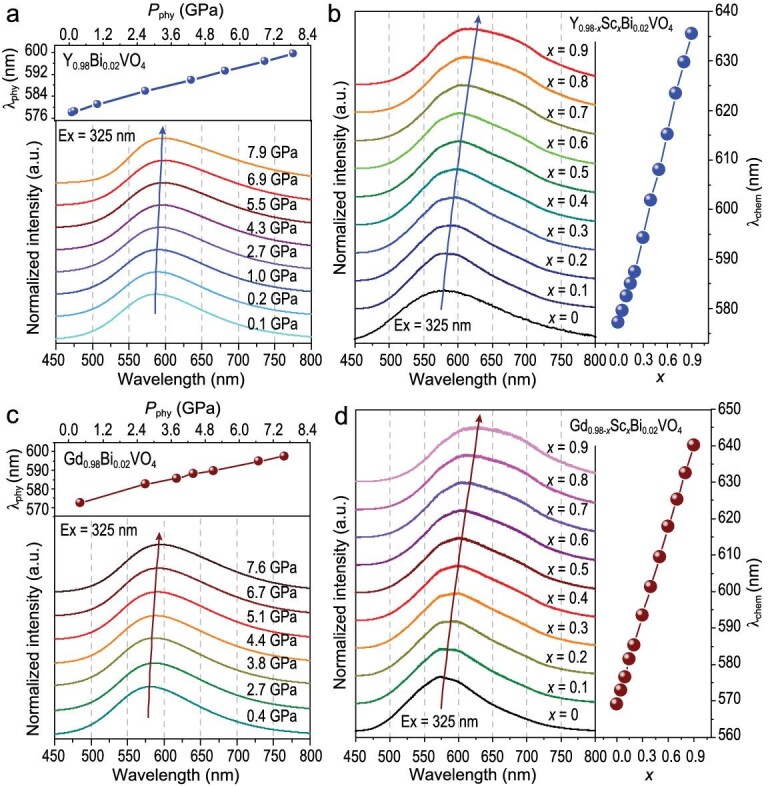
The emission spectra and the position shift (the maximum emission intensity) of Y_0.98_Bi_0.02_VO_4_ (a) and Gd_0.98_Bi_0.02_VO_4_ (c) under high pressure. The emission spectra and the position shift (the maximum emission intensity) of Y_0.98-_*_x_*Sc*_x_*Bi_0.02_VO_4_ (b) and Gd_0.98-_*_x_*Sc*_x_*Bi_0.02_VO_4_ (d) with different ion substitution concentrations *x*.

Figure 4a presents the *in-situ* pressure-dependent normalized emission spectra of Y_0.98_Bi_0.02_VO_4_ up to 7.9 GPa, and the position shift of emission spectra under *P*_phy_ is shown in the inset of Fig. 4a. The yellow-green light from Bi^3+^ was recorded in 480–800 nm upon an indirect excitation via the charge transfer (CT) state at 325 nm. The excitation spectra of the Y_0.98-_*_y_*Bi*_y_*VO_4_ consist of overlapping transitions (CT) of different origins: one CT occurs within VO_4_^3−^ molecular units, and the other takes place between Bi^3+^ and V^5+^ (Fig. S3). Upon pressing, the emission band exhibits a noticeable red shift, with maximum peak intensity centralized at *λ*_phy_ of 585 and 610 nm (*Δλ*_phy_ = 25 nm) for ambient pressure (Atm.) and 7.9 GPa, respectively. Similar phenomena are also observed in Gd_0.98_Bi_0.02_VO_4_, where the *λ*_phy_ at maximum intensity shifts from 575 to 598 nm (*Δλ*_phy_ = 23 nm) when *P*_phy_ increases from Atm. to 7.6 GPa (Fig. 4c). Fluorescence photographs of Y_0.98_Bi_0.02_VO_4_ and Gd_0.98_Bi_0.02_VO_4_ in a microscopic system (visual field: ∼200 *μ*m) excited at 330 nm (Xenon lamp) are displayed in Fig. S8a and b. These results show that fluorescence intensity decreases and the luminescence change (Y_0.98_Bi_0.02_VO_4_: yellow → orange; Gd_0.98_Bi_0.02_VO_4_: green-yellow → yellow) can be observed by the naked eyes under *P*_phy_. According to previous studies on Bi^3+^ in YVO_4_ and GdVO_4_ [52], the electronic transition from the excited ^3^P_1_ state to the ground ^1^S_0_ state of Bi^3+^ is responsible for the emission band. The above *P*_phy_-induced spectral red shift is commonly observed and ascribed to the splitting energy of the crystal field belonging to the ^3^P_1_ → ^1^S_0_ transition of Bi^3+^, which is locally governed by the deformation of the *RE*O_8_ dodecahedral group under pressure.

Parallel emission spectra of Y_0.98-_*_x_*Sc*_x_*Bi_0.02_VO_4_ and Gd_0.98-_*_x_*Sc*_x_*Bi_0.02_VO_4_ (0 ≤ *x* ≤ 0.9) were collected to monitor the *P*_chem-_*_Δ_* effect. With the increase of *x*, the emission band shows an obvious red shift (Fig. 4b and d; Fig. S5b and c), which is consistent with the effect of *P*_phy_. The concurrent red shift demonstrates that optically correlated *P*_chem-_*_Δ_* effectively simulates the *P*_phy_ at the local structure scale (*RE*O_8_) as observed in this work. The peak position (*λ*_chem_) at maximum intensity shifts from ∼577 to 636 nm at *x* = 0 and 0.9 in Y_0.98-_*_x_*Sc*_x_*Bi_0.02_VO_4_, over a tuning range of ∼59 nm, which is consistent with the literature report [61]. Comparably, the increase of the *x* value from 0 to 0.9 in Gd_0.98-_*_x_*Sc*_x_*Bi_0.02_VO_4_ leads to a red shift emission of ∼71 nm. In these cases, the red shift of Bi^3+^ emission is mainly due to the shortened *RE*/Sc−O bond lengths during the modulation from *RE*_0.98_Bi_0.02_VO_4_ to *RE*_0.08_Sc_0.9_Bi_0.02_VO_4_, dominated by crystal field strength around the luminescence center of the Bi^3+^ ion [61]. The stronger crystal field leads to a drop in the Bi^3+^:^3^P_1_ level. This results in a red shift and significant broadening of the Bi^3+^ emission [61,62]. Luminescence photographs (Fig. S8c and d) of samples with different *x* were taken in the black box with a 325 nm excitation light source. The photos demonstrate that luminescence intensity has no outstanding change as *x* rises, and the luminescence trend of Y_0.98-_*_x_*Sc*_x_*Bi_0.02_VO_4_ from yellow to orange light correlates to the red shift of the emission band (Fig. S8c), which is comparable to the change that occurred under *P*_phy_ (Fig. S8a). For Gd_0.98-_*_x_*Sc*_x_*Bi_0.02_VO_4_ luminescence evolution is from green to green-yellow light (Fig. S8d), corresponding to the larger red shift in the emission band under *P*_chem-_*_Δ_* (Fig. S8b). It should be noted that the photos under *P*_phy_ and *P*_chem-_*_Δ_* were taken with different methods and light sources (330 *vs.* 325 nm), the luminescence of the Gd-samples is around the color crossover and exhibits noticeably distinct colors.

#### Calibration of local chemical pressure

**Figure 5.**
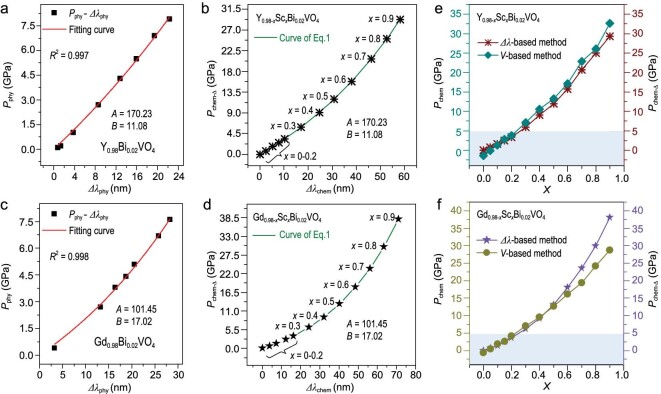
Pressure-dependent spectra shift (*Δλ*_phy_) of Y_0.98_Bi_0.02_VO_4_ (a) and Gd_0.98_Bi_0.02_VO_4_ (c) fitted by the Mao–Bell equation Eq (1). The curve (green) of Eq (1), reflecting the relationship between the concentration-dependent spectra shift (*Δλ*_chem_) and the local chemical pressure (*P*_chem-_*_Δ_*) of Y_0.98-_*_x_*Sc*_x_*Bi_0.02_VO_4_ (b) and Gd_0.98-_*_x_*Sc*_x_*Bi_0.02_VO_4_ (d). The relationship between *P*_chem_/*P*_chem-_*_Δ_* and *x* (the ion substitution concentration) of Y_0.98-_*_x_*Sc*_x_*Bi_0.02_VO_4_ (e) and Gd_0.98-_*_x_*Sc*_x_*Bi_0.02_VO_4_ (f) from *V* and *Δλ-*based method.

The calibration of traced *P*_chem-_*_Δ_* can be realized by spectra shift *Δλ* in Fig. 5, which bridges the *P*_chem-_*_Δ_* with *P*_phy_. According to previous reports [62–64], utilizing the parabolic, exponential, or linear equation to fit the pressure data versus *Δλ* can satisfy the *R*-squares approaching 1. Here, the Mao–Bell equation (Eq., exponential) [1] can be used to define the relationship between pressure *P* and spectral shift *Δλ*[64].


(1) \begin{eqnarray*} P = \frac{A}{B}\left\{ {{{\left[ {1 + \left( {\frac{{\Delta \lambda }}{{{\lambda }_0}}} \right)} \right]}}^B - 1} \right\}. \end{eqnarray*}

The *P*_phy_-induced spectral shift *Δλ*_phy_ of Y_0.98_Bi_0.02_VO_4_ is listed in Table 1. Eq. (1) was used to fit the *P*_phy_–*Δλ*_phy_ data (Fig. 5a) of Y_0.98_Bi_0.02_VO_4_. Here *P*_phy_ is read from DAC, *λ*_0_ = 577.2 nm is the wavelength at 1 bar, *λ*_phy_ is obtained by fitting the peak form function, *Δλ*_phy_ is the wavelength shift measured at *P*_phy_ referenced to *λ*_0_. The fitted *A* and *B* are 170.23 GPa and 11.08, respectively. *P*_chem-_*_Δ_* can be derived by Eq. (1) with *R*^2^ = 0.997 (Table 1). The obtained *P*_chem-_*_Δ_ vs. Δλ* data points and the fitting curve by Eq. (1) for Y_0.98-_*_x_*Sc*_x_*Bi_0.02_VO_4_ are shown in Fig. 5b. Similarly, the *P*_chem-_*_Δ_ vs. Δλ* based calibration for Gd_0.98-_*_x_*Sc*_x_*Bi_0.02_VO_4_ results in *A* = 101.45 GPa, *B* = 17.02, *λ*_0_ = 569.2 nm, and *R*^2^ = 0.998 (Fig. 5c and d). The above optical-probe strategy for the first time builds a bridge between *P*_phy_ and *P*_chem-_*_Δ_*, and makes it possible to get insights into *P*_chem_ at spatially higher resolution into chemical-bond evolution.

**Table 1.** Experimental data, fitting results and relative errors of related parameters.

Y_0.98_Bi_0.02_VO_4_						Y_0.98-_*_x_*Sc*_x_*Bi_0.02_VO_4_					
HP-SPXD		HP-OM									
*P*_phy_ (GPa)	*V*_phy_ (Å^3^)	*P*_phy_ (GPa)	*λ*_phy_ (nm)	*Error* (nm)	*x*	PXD *Vx* (Å^3^)	*V*-based *P*_chem_ (GPa)	OM *λ*_chem_ (nm)	*Error* (nm)	*Δλ*-based *P*_chem_*_-Δ_* (GPa)	*RD*
1.3	79.0	0.1	578.0	0.3	0	79.9	−1.4	577.2	0.2	0	1.4
1.9	78.8	0.2	578.6	0	0.05	79.4	−0.1	579.6	0.2	0.7	0.8
3.3	78.4	1.0	581.0	0.2	0.1	79.0	1.3	582.5	0.1	1.6	0.4
4.6	78.0	2.7	585.8	0.3	0.15	78.6	2.8	585.0	0.1	2.5	0.3
6.3	77.5	4.3	590.0	0.1	0.2	78.2	3.8	587.5	0	3.3	0.5
7.3	77.2	5.5	593.2	0.1	0.3	77.3	7.1	594.3	0.1	5.9	1.2
		6.9	596.6	0.1	0.4	76.4	10.6	601.9	0.1	9.1	1.5
		7.9	599.5	0.1	0.5	75.7	13.2	608.0	0	12.0	1.2
					0.6	74.7	17.1	615.2	0.1	15.8	1.4
					0.7	73.5	22.8	623.4	0.2	20.7	2.1
					0.8	72.8	26.1	629.8	0.2	25.0	1.1
					0.9	71.5	32.7	635.5	0.3	29.3	3.4
**Gd_0.98_Bi_0.02_VO_4_**						**Gd_0.98-_*_x_*Sc*_x_*Bi_0.02_VO_4_**					
**HP-SPXD**		**HP-OM**									
***P*_phy_ (GPa)**	***V*_phy_ (Å^3^)**	***P*_phy_ (GPa)**	***λ*_phy_ (nm)**	***Error* (nm)**	***x***	**PXD *Vx* (Å^3^)**	***V*-based *P*_chem_ (GPa)**	**OM *λ*_chem_ (nm)**	***Error* (nm)**	***Δλ*-based *P*_chem_*_-Δ_* (GPa)**	***RD***
1.4	81.7	0.4	572.5	0.2	0	82.7	−0.6	569.2	0.1	0	0.6
1.8	81.4	2.7	582.4	0	0.05	82.1	0.4	573.0	0	0.7	0.3
2.5	80.9	3.8	585.6	0.1	0.1	81.4	1.9	576.5	0	1.4	0.5
3.5	80.5	4.4	588.0	0.1	0.15	81.0	2.6	581.5	0.1	2.6	0
4.8	80.1	5.1	589.8	0.1	0.2	80.3	4.1	585.4	0.1	3.6	0.5
5.9	79.6	6.7	595.0	0	0.3	79.0	7.0	593.5	0.1	6.2	0.8
7.0	79.0	7.6	597.5	0	0.4	78.0	9.5	601.3	0.1	9.2	0.3
					0.5	76.7	12.7	609.5	0.2	13.1	0.4
					0.6	75.5	16.1	617.8	0.2	18.1	2.0
					0.7	74.4	24.3	625.3	0.2	23.6	4.0
					0.8	72.9	26.6	632.5	0.2	29.9	5.6
					0.9	71.7	28.7	640.2	0.3	38.1	9.4

**HP-SPXD** is the synchrotron radiation experiment under high pressure. **HP-OM** is an optical measurement under high pressure. **OM** is the optical measurement. **PXD** is a powder X-ray diffraction experiment. ***P*_phy_** is physical pressure in **HP-SPXD** and **HP-OM. *V*_phy_** is the unit cell volume in **HP-SPXD** from the refined result of *RE*_0.98_Bi_0.02_VO_4_, ***V_x_*** is the unit cell volume in PXD from the refined result of *RE*_0.98-_*_x_*Sc*_x_*Bi_0.02_VO_4_ with different ion substitution concentration *x*, ***P*_chem_** is chemical pressure from the *V*-based calibration method. ***λ*_phy_** is the position of the maximum emission intensity in **HP-OM** of *RE*_0.98_Bi_0.02_VO_4_, ***λ*_chem_** is the position of the maximum emission intensity in **OM** of *RE*_0.98-_*_x_*Sc*_x_*Bi_0.02_VO_4_ with different ion substitution concentration *x*, ***P*_chem-_*_Δ_*** is local chemical pressure from the *Δλ*-based calibration method. ***RD*** is the relative error between the *Δλ*-based calibration method and the *V*-based calibration method. ***Error*** comes from fluorescence peak fitting.

### Advantages and practicability of the ***Δλ***-based method

Based on the crystal field splitting (CFS) theory [65,66], the energy difference between the highest and lowest energy levels in a crystal is recognized as the CFS energy. This energy is influenced by multiple factors, including the bond length between the activator and ligand, the covalent degree, the coordination environment, and the symmetry of the position of the activator ion. Hence the *Δλ*-based calibration of *P*_chem-_*_Δ_* can reflect the effect of pressure (physically and chemically) on the crystal field scale covered by the above factors. The *P*_chem-_*_Δ_* can be calibrated and described from a new perspective by breaking the confinement of the cell-volume dimension. It is possible to calibrate chemical pressure using pluralistic optical parameters (*O*_chem_ ≈ *O*_phy_) other than *Δλ*, such as intensity ratio, fluorescence lifetime, and so on, which make this methodology universal. In this case, the optical probe Bi^3+^ is the activator ion of which the emission spectra are impacted by the crystal field evolution of *RE*_0.98-_*_x_*Sc*_x_*Bi_0.02_VO_4_ (0 ≤ *x* ≤ 0.9) under *P*_phy_ or *P*_chem_. The consistent red shift in the emission spectra illustrates that *P*_chem-_*_Δ_* effectively simulates the crystal field evolution under *P*_phy_. From previous local structural analysis, there are two points of similarity under both *P*_phy_ and *P*_chem-_*_Δ_*, these are (1) the compression of the local *RE*O_8_ group and (2) the shortening of *RE*-O2 bond length, indicating that the red shift of emission spectra is congeneric, and the other factors hardly affect the signal concerned. Compared to the low sensitivity of the *RE*−O1 bond length under *P*_phy_, the *RE*−O1 bond length is less rigid under *P*_chem-_*_Δ_*, suggesting that the dodecahedron is distorted in a different manner under both pressures. The evolution of the VO_4_ group is also different in the two-type pressures. Owing to the phase transition, *P*_chem_ and *P*_chem-_*_Δ_* calibration is only based on monitoring data over the 0–8 GPa range. The above differences cause an error (*D*) between *P*_phy_ and *P*_chem-_*_Δ_*. In this work, we established the connection between *P*_phy_ and *P*_chem-_*_Δ_* by assuming *P*_phy_/*O*_phy_ ≈ *P*_chem-_*_Δ_*/*O*_chem_ based on the congeneric local structure evolution. Actually, it is *P*_chem_ + *D* = *P*_phy_, and the *D* value depends on the total error factors.

The error factor *D* in the *V*-based method correlates to the cell-dimension variation. In *RE*_0.98-_*_x_*Sc*_x_*Bi_0.02_VO_4_, the change of *V* and (*a, c*) is analogous under both pressures, demonstrating that the *D* value is very low. Therefore, the *P*_chem_ value can be used as a reference to compare the error of the results from the *Δλ*-based calibration method, which is the relative error (*RD*). In local structures, the different evolution under *P*_phy_ and *P*_chem-_*_Δ_* could result in increased *D* value from the *Δλ*-based calibration. Figure 5e and f compare the outcomes of the *V*- and *Δλ* (optical probe)-based calibration methods. The *RD* tends to be zero below 6 GPa (*x*<0.3), indicating that the chemical pressure in this region can effectively simulate *P*_phy_ at both *V*- and crystal-field scales. It supports the idea that the spectral red shift does result from the shortening of *RE*−O bond length under pressure rather than other factors. The ‘impure’ nature of chemical pressure emerges as *x* increases, in which the corresponding *RD* value raises, and the structural (including cell dimension and local structure) consistency between chemical and physical pressures gets weakened. Accordingly, substitution with a larger ionic-size difference to the host is highly desirable to introduce considerable *P*_chem_ at low ion substitution concentrations, which, in other words, can achieve the interception of metastable physical characteristics with a low *D* value, rendering *P*_chem-_*_Δ_* and *P*_phy_ more similar.

## CONCLUSIONS

In summary, the developed methodology experimentally realizes the calibration of local chemical pressure by a site-selective optical probe, building a solid connection (scale-bar) between physical and local chemical pressure as exemplified by Bi^3+^-traced *RE*VO_4_ (*RE* = Y or Gd). Exerting chemical pressure by site-selective small ion substitution (Sc^3+^ → *RE*^3+^) in *RE*_0.98-_*_x_*Sc*_x_*Bi_0.02_VO_4_ effectively mimics the anisotropic cell compression under physical pressure in the parent *RE*_0.98_Bi_0.02_VO_4_. The red shift (*Δλ*) of Bi^3+^ emission spectra on-site senses the alteration of the crystal field under physical/local chemical pressure. This *Δλ*-based approach not only pictures the local chemical pressure in the crystal field dimension beyond the widely used unit-cell volume criteria but also decomposes the chemical pressure into a higher spatial resolution (local-motif scale and chemical-bond evolution). This optical-probe calibration of local chemical pressure forms a new perspective for chemical stabilization of the high-pressure metastable phase, significantly guides the exploration of chemical pressure limitation, and sheds light on the calibration of locally in-plane/out-of-plane external chemical pressure, which will be the focus on on-site portrayal of the interfacial strain/stress and equivalent physical pressure.

## METHODS

### Synthesis

All samples were synthesized by solid-state reaction. Stoichiometric mixture of the raw materials Y_2_O_3_ (Macklin 99.99%), Gd_2_O_3_ (Aladdin 99.99%), Sc_2_O_3_ (Aladdin 99.99%), NH_4_VO_3_ (Macklin 99.99%), and Bi_2_O_3_ (Aladdin 99.99%) was sufficiently ground and pelletized before being placed in an alumina crucible for furnace heating. Two different calcination programs (heating rate of 5^o^C/min; *x* = 0–0.9, *y* = 0–0.3) were applied: (1) Calcined at 1000^o^C for 6 h in the air to prepare Y_0.98-_*_x-y_*Sc*_x_*Bi*_y_*VO_4_; (2) Calcined at 1200^o^C for 6 h in air with intermediate grinding to prepare Gd_0.98-_*_x-y_*Sc*_x_*Bi*_y_*VO_4_. After naturally cooling to room temperature, the samples were milled into powders for the following measurements.

### Characterizations

Phase purity and identification of the samples (*RE*_0.98-_*_x_*Sc*_x_*Bi_0.02_VO_4_) were analyzed by powder X-ray diffraction (PXD) on a SmartLab SE diffractometer (Rigaku, Japan) using Cu-K*α* (*λ* = 0.15 418 nm, 40 kV, 40 mA) radiation. The PXD data used for detailed structure analyses were collected over a 2*θ* range of 10–120° using a step size of 0.01° with 0.5^o^/min. Room-temperature SPXD data were recorded on the beamline BL15U1 (*λ* = 0.6199 Å) at the Shanghai Synchrotron Radiation Facility (SSRF) for *RE*_0.98_Bi_0.02_VO_4_ in a DAC with silicone oil as PTM under high-pressure. Rietveld refinements were performed with the TOPAS software package [67]. The fluorescent images under high pressure were obtained by a Nikon Eclipse Ti2 microscope equipped with a digital color camera. The fluorescent images of powder samples (*RE*_0.98-_*_x_*Sc*_x_*Bi_0.02_VO_4_) with different substitution concentrations were taken in an ultraviolet camera-obscura. All photoluminescence/Raman spectra exciting with the ultraviolet/633 nm laser were recorded using a Raman spectrometer (inVia Renishaw) equipped with a microscope system (Leica, Germany). Physical pressure was generated in a symmetric DAC (culet diameter of 500 *μ*m). The powder sample was loaded into the holes (diameter ca. 200 *μ*m) of a T301 steel gasket pre-indented to a thickness of 50 *μ*m. A small ruby ball was inserted into the sample compartment for *in-situ* pressure calibration according to the *R*_1_ ruby fluorescence method [36].

### Theoretical calculations

The local structures of YVO_4_ and GdVO_4_ under pressure were simulated by DFT calculations performed in the Vienna *Ab initio* Simulation Package (VASP) and the projector-augmented wave method [68]. Generalized gradient approximation (GGA) implemented in the revised Perdew − Burke − Ernzerhof for solid (PBEsol) method [69] was employed for constant-volume structural optimization of YVO_4_ and GdVO_4_. The plane-wave base set was employed with a cutoff energy of 520 eV. All the forces were converged to 0.01 eV/Å. A *Γ*-centered *k*-point grid of 9 × 9 × 9 was used for sampling in the Brillouin zone. The pressure for each volume was calculated by fitting the constant volume into the second-order Birch-Murnaghan equation of state [70], the parameters of which resulted from the fitting of pressures versus volumes refined from SPXD data. Bond lengths and polyhedral volumes were read and calculated from the optimized structures, respectively.

## Supplementary Material

nwad190_Supplemental_File

## References

[1] McMillan PF . New materials from high-pressure experiments. Nat Mater 2002; 1: 19–25. 10.1038/nmat716 12618843

[2] Zhang L , Wang Y, Lv J et al. Materials discovery at high pressures. Nat Rev Mater 2017; 2: 17005. 10.1038/natrevmats.2017.5

[3] Xiao G , Geng T, Zou B. Emerging functional materials under high pressure toward enhanced properties. ACS Mater Lett 2020; 2: 1233–9. 10.1021/acsmaterialslett.0c00329

[4] Zhao D , Wang M, Xiao G et al. Thinking about the development of high-pressure experimental chemistry. J Phys Chem Lett 2020; 11: 7297–306. 10.1021/acs.jpclett.0c02030 32787316

[5] Fu Z , Wang K, Zou B. Recent advances in organic pressure-responsive luminescent materials. Chin Chem Lett 2019; 30: 1883–94. 10.1016/j.cclet.2019.08.041

[6] Snider E , Dasenbrock-Gammon N, McBride R et al. Room-temperature superconductivity in a carbonaceous sulfur hydride. Nature 2020; 586: 373–7. 10.1038/s41586-020-2801-z 33057222

[7] Errea I , Belli F, Monacelli L et al. Quantum crystal structure in the 250-Kelvin superconducting lanthanum hydride. Nature 2020; 578: 66–9. 10.1038/s41586-020-1955-z 32025016

[8] Sun L , Chen X-J, Guo J et al. Re-emerging superconductivity at 48 Kelvin in iron chalcogenides. Nature 2012; 483: 67–9. 10.1038/nature10813 22367543

[9] Dong X , Oganov AR, Goncharov AF et al. A stable compound of helium and sodium at high pressure. Nat Chem 2017; 9: 440–5. 10.1038/nchem.2716 28430195

[10] Zhang W , Oganov AR, Goncharov AF et al. Unexpected stable stoichiometries of sodium chlorides. Science 2013; 342: 1502–5. 10.1126/science.1244989 24357316

[11] Tian Y , Xu B, Yu D et al. Ultrahard nanotwinned cubic boron nitride. Nature 2013; 493: 385–8. 10.1038/nature11728 23325219

[12] Li M-R , Retuerto M, Stephens PW et al. Low-temperature cationic rearrangement in a bulk metal oxide. Angew Chem Int Ed 2016; 55: 9862–7. 10.1002/anie.201511360 27203790

[13] Li M-R , Retuerto M, Walker D et al. Magnetic-structure-stabilized polarization in an above-room-temperature ferrimagnet. Angew Chem Int Ed 2014; 53: 10774–8. 10.1002/anie.201406180 25131837

[14] Shi Y , Guo Y, Wang X et al. A ferroelectric-like structural transition in a metal. Nat Mater 2013; 12: 1024–7. 10.1038/nmat3754 24056805

[15] Tse JS . A chemical perspective on high pressure crystal structures and properties. Natl Sci Rev 2020; 7: 149–69. 10.1093/nsr/nwz144 34692029 PMC8289026

[16] Huppertz H . New synthetic discoveries via high-pressure solid-state chemistry. Chem Commun 2011; 47: 131–40. 10.1039/C0CC02715D 20859577

[17] McMillan PF . High pressure synthesis of solids. Curr Opin Solid State Mater Sci 1999; 4: 171–8. 10.1016/S1359-0286(99)00013-3

[18] Huppertz H . Multianvil high-pressure /high-temperature synthesis in solid state chemistry. Cheminform 2004; 219: 330–8. 10.1524/zkri.219.6.330.34633

[19] Ke F , Dong HN, Chen YB et al. Decompression-driven sperconductivity enhancement in In_2_Se_3_. Adv Mater 2017; 29: 1701983. 10.1002/adma.201701983 28692745

[20] Lin K , Li Q, Yu RZ et al. Chemical pressure in functional materials. Chem Soc Rev 2022; 51: 5351–64. 10.1039/D1CS00563D 35735127

[21] Fournier J-M . Chemical pressure in actinide systems. Physica B 1993; 190: 50–4. 10.1016/0921-4526(93)90441-8

[22] Dawley NM , Marksz EJ, Hagerstrom AM et al. Targeted chemical pressure yields tuneable millimetre-wave dielectric. Nat Mater 2020; 19: 176–81. 10.1038/s41563-019-0564-4 31873229

[23] Chen XL , Eysel E. The stabilization of *β*-Bi_2_O_3_ by CeO_2_. J Solid State Chem 1996; 127: 128–30. 10.1006/jssc.1996.0367

[24] Zhang L , Chen J, Fan L et al. Giant polarization in super-tetragonal thin films through interphase strain. Science 2018; 361: 494–7. 10.1126/science.aan2433 30072536

[25] Shi G , Chen L, Yang Y et al. Two-simensional Na–Cl crystals of unconventional stoichiometries on graphene surface from dilute solution at ambient conditions. Nat Chem 2018; 10: 776–9. 10.1038/s41557-018-0061-4 29736004

[26] Son JY , Lee G, Jo M-H et al. Heteroepitaxial ferroelectric ZnSnO_3_ thin film. J Am Chem Soc 2009; 131: 8386–7. 10.1021/ja903133n 19476356

[27] Li M-R , Adem U, McMitchell SRC et al. A polar corundum oxide displaying weak ferromagnetism at room temperature. J Am Chem Soc 2012; 134: 3737–47. 10.1021/ja208395z 22280499 PMC3693400

[28] Gich M , Fina I, Morelli A et al. Multiferroic iron oxide thin films at room temperature. Adv Mater 2014; 26: 4645–52. 10.1002/adma.201400990 24831036

[29] Rao BN , Yasui S, Han Y et al. Redox-based multilevel resistive switching in AlFeO_3_ thin-film heterostructures. ACS Appl Electron Mater 2020; 2: 1065–73. 10.1021/acsaelm.0c00083

[30] Li Z , Cho Y, Li X et al. New mechanism for ferroelectricity in the perovskite Ca_2–_*_x_*Mn*_x_*Ti_2_O_6_ synthesized by spark plasma sintering. J Am Chem Soc 2018; 140: 2214–20. 10.1021/jacs.7b11219 29334457

[31] Paglione J , Greene RL. High-temperature superconductivity in iron-based materials. Nat Phys 2010; 6: 645–58. 10.1038/nphys1759

[32] Kimber SAJ , Kreyssig A, Zhang Y-Z et al. Similarities between structural distortions under pressure and chemical doping in superconducting BaFe_2_As_2_. Nat Mater 2009; 8: 471–5. 10.1038/nmat2443 19404240

[33] Rotter M , Pangerl M, Tegel M et al. Superconductivity and crystal structures of (Ba_1−_*_x_*K*_x_*)Fe_2_As_2_ (*x* = 0–1). Angew Chem Int Ed 2008; 47: 7949–52. 10.1002/anie.200803641 18780403

[34] Ren Z , Tao Q, Jiang S et al. Superconductivity induced by phosphorus doping and its coexistence with ferromagnetism in EuFe_2_(As_0.7_P_0.3_)_2_. Phys Rev Lett 2009; 102: 137002. 10.1103/PhysRevLett.102.137002 19392395

[35] Sun R , Jin S, Deng J et al. Chemical pressure boost record-high superconductivity in van der Waals materials FeSe_1−_*_x_*S*_x_*. Adv Funct Mater 2021; 31: 2102917. 10.1002/adfm.202102917

[36] Mao HK , Xu J, Bell PM. Calibration of the Ruby pressure gauge to 800 Kbar under quasi-hydrostatic conditions. J Geophys Res 1986; 91: 4673–6. 10.1029/JB091iB05p04673

[37] Wang Z , Liu Y, Bi Y et al. Hydrostatic pressure and temperature calibration based on phase diagram of bismuth. High Pressure Res 2012; 32: 167–75. 10.1080/08957959.2012.677950

[38] Das P , Kanchanavatee N, Helton JS et al. Chemical pressure tuning of URu_2_Si_2_ via isoelectronic substitution of Ru with Fe. Phys Rev B 2015; 91: 85122. 10.1103/PhysRevB.91.085122

[39] Mizuguchi Y , Paris E, Sugimoto T et al. The effect of RE substitution in layered REO_0.5_F_0.5_BiS_2_: chemical pressure, local disorder and superconductivity. Phys Chem Chem Phys 2015; 17: 22090–6. 10.1039/C5CP03750F 26234627

[40] Han Y , Zeng Y, Hendrickx M et al. Universal A-cation splitting in LiNbO_3_-type structure driven by intrapositional multivalent coupling. J Am Chem Soc 2020; 142: 7168–78. 10.1021/jacs.0c01814 32216316

[41] Fredrickson DC . Electronic packing frustration in complex intermetallic structures: the role of chemical pressure in Ca_2_Ag_7_. J Am Chem Soc 2011; 133: 10070–3. 10.1021/ja203944a 21619054

[42] Lu X , Stoumpos C, Hu Q et al. Regulating off-centering distortion maximizes photoluminescence in halide perovskites. Natl Sci Rev 2021; 8: nwaa288. 10.1093/nsr/nwaa288 34691729 PMC8433095

[43] Li Y , Qin L, Huang SY et al. Enhanced magnetocaloric performances and tunable martensitic transformation in Ni_35_Co_15_Mn_35-_*_x_*Fe*_x_*Ti_15_ all-*d*-metal heusler alloys by chemical and physical pressures. Sci China Mater 2022; 65: 486–93. 10.1007/s40843-021-1747-3

[44] Zhou X , Yang J, Zhu C et al. Robust yellow-violet pigments tuned by site-selective manganese chromophores. Inorg Chem 2021; 60: 11579–90. 10.1021/acs.inorgchem.1c01568 34259522

[45] Richet P , Xu J-A, Mao H-K. Quasi-hydrostatic compression of Ruby to 500 Kbar. Phys Chem Miner 1988; 16: 207–11. 10.1007/BF00220687

[46] Jayaraman A , Kourouklis GA, Espinosa GP et al. A high-pressure Raman study of yttrium vanadate (YVO_4_) and the pressure-induced transition from the zircon-type to the scheelite-type structure. J Phys Chem Solids 1987; 48: 755–9. 10.1016/0022-3697(87)90072-2

[47] Yue B , Hong F, Merkel S et al. Deformation behavior across the zircon-scheelite phase transition. Phys Rev Lett 2016; 117: 135701. 10.1103/PhysRevLett.117.135701 27715087

[48] Hazen RM , Finger LW, Mariathasan JWE. High-pressure crystal chemistry of scheelite-type tungstates and molybdates. J Phys Chem Solids 1985; 46: 253–63. 10.1016/0022-3697(85)90039-3

[49] Awater RHP , Dorenbos P. Towards a general concentration quenching model of Bi^3+^ luminescence. J Lumin 2017; 188: 487–9. 10.1016/j.jlumin.2017.05.011

[50] Cao RP , Quan GJ, Shi ZH et al. A double perovskite Ca_2_MgWO_6_:Bi^3+^ yellow-emitting phosphor: synthesis and luminescence properties. J Lumin 2017; 181: 332–6. 10.1016/j.jlumin.2016.09.046

[51] Begum R , Parida MR, Abdelhady AL et al. Engineering interfacial charge transfer in CsPbBr_3_ perovskite nanocrystals by heterovalent doping. J Am Chem Soc 2017; 139: 731–7. 10.1021/jacs.6b09575 27977176

[52] Li JH , Liu J, Yu XB. Synthesis and luminescence properties of Bi^3+^-doped YVO_4_ phosphors. J Alloys Compd 2011; 509: 9897–900. 10.1016/j.jallcom.2011.07.079

[53] Shannon RD . Revised effective ionic radii and systematic studies of interatomic distances in halides and chalcogenides. Acta Cryst A 1976; A32: 751–67. 10.1107/S0567739476001551

[54] Wang X , Loa I, Syassen K et al. Structural properties of the zircon- and scheelite-type phases of YVO_4_ at high pressure. Phys Rev B 2004; 70: 06419. 10.1103/PhysRevB.70.064109

[55] Shikama N , Sakishita Y, Nabeshima F et al. Positive and negative chemical pressure effects investigated in electron-doped FeSe films with an electric-double-layer structure. Phys Rev B 2021; 104: 094512. 10.1103/PhysRevB.104.094512

[56] Marqueno T , Errandonea D, Pellicer-Porres J et al. High-pressure polymorphs of gadolinium prthovanadate: X-ray diffraction, Raman spectroscopy, and ab initio calculations. Phys Rev B 2019; 100: 064106. 10.1103/PhysRevB.100.064106

[57] Manjon FJ , Rodriguez-Hernandez P, Munoz A et al. Lattice dynamics of YVO_4_ at high pressures. Phys Rev B 2010; 81: 075202. 10.1103/PhysRevB.81.075202

[58] Zhou X , Zhao MH, Yang J et al. Chemical pressure enlarged camouflage color zone in Mn(IV)-activated yellow-green pigments. Mater Today Chem 2022; 25: 100902.

[59] Li MR , Stephens PW, Retuerto M et al. Designing polar and magnetic oxides: Zn_2_FeTaO_6_ - in search of multiferroics. J Am Chem Soc 2014; 136: 8508–11. 10.1021/ja502774v 24841411

[60] Li MR , Hodges JP, Retuerto M et al. Mn_2_MnReO_6_: synthesis and magnetic structure determination of a new transition-metal-only double perovskite canted antiferromagnet. Chem Mater 2016; 28: 3148–58. 10.1021/acs.chemmater.6b00755

[61] Kang FW , Zhang HS, Wondraczek L et al. Band-gap modulation in single Bi^3+^-doped yttrium-scandium-niobium vanadates for color tuning over the whole visible spectrum. Chem Mater 2016; 28: 2692–703. 10.1021/acs.chemmater.6b00277

[62] Wang Y , Seto T, Ishigaki K et al. Pressure-driven Eu^2+^-doped BaLi_2_Al_2_Si_2_N_6_: a new color tunable narrow-band emission phosphor for spectroscopy and pressure sensor applications. Adv Funct Mater 2020; 30: 2001384. 10.1002/adfm.202001384

[63] Runowski M , Wozny P, Stopikowska N et al. Optical pressure sensor based on the emission and excitation band width (fwhm) and luminescence shift of Ce^3+^-doped fluorapatite-high-pressure sensing. ACS Appl Mater Interfaces 2019; 11: 4131–8. 10.1021/acsami.8b19500 30615827

[64] Mao HK , Bell PM, Shaner JW et al. Specific volume measurements of Cu, Mo, Pd, and Ag and calibration of the Ruby R1 fluorescence pressure gauge from 0.06 to 1 Mbar. J Appl Phys (USA) 1978; 49: 3276–83. 10.1063/1.325277

[65] Shang MM , Liang SS, Qu NR et al. Influence of anion/cation substitution (Sr^2+^ → Ba^2+^, Al^3+^ → Si^4+^, N^3−^ → O^2−^) on phase transformation and luminescence properties of Ba_3_Si_6_O_15_:Eu^2+^ phosphors. Chem Mater 2017; 29: 1813–29. 10.1021/acs.chemmater.6b05493

[66] Gerloch M , Slade RC. Ligand-Field Parameters. Cambridge: Cambridge University Press, 1973, 31–6.

[67] Bruker AXS . Topas Academic: General Profile and Structure Analysis Software for Powder Diffraction Data. Karlsruhe: User Manual Bruker AXS, 2012.

[68] Kresse G , Joubert D. From ultrasoft pseudopotentials to the projector augmented-wave method. Phys Rev B 1999; 59: 1758–75. 10.1103/PhysRevB.59.1758

[69] Csonka GI , Perdew JP, Ruzsinszky A et al. Assessing the performance of recent density functionals for bulk solids. Phys Rev B 2009; 79: 155107. 10.1103/PhysRevB.79.155107

[70] Birch F . Finite elastic strain of cubic crystals. Phys Rev 1947; 71: 809–24. 10.1103/PhysRev.71.809

