# Supplementary material for: Calibration of local chemical pressure by optical probe
Source: Natl Sci Rev. 2023 Jul 10;10(9):nwad190. doi: 10.1093/nsr/nwad190 (PMC10411671; doi:10.1093/nsr/nwad190)
Supplement: nwad190_Supplemental_File [file nwad190_supplemental_file.pdf]

Supporting Information

Calibration of Local Chemical Pressure by Optical Probe

Xiao Zhou,[a, #] Mei-Huan Zhao,[a, #] Shan-Ming Yao,[a] Hongliang Dong,[b] Yonggang Wang,[c] Bin Chen,[b] Xianran Xing[d,*] Man-Rong Li,[a, e,*]

[a]Key Laboratory of Bioinorganic and Synthetic Chemistry of Ministry of Education, School of Chemistry, Sun Yat-Sen University, Guangzhou 510275, China.

[b]Center for High Pressure Science and Technology Advanced Research, Shanghai 201203, China

[c]School of Materials Science and Engineering, Peking University, Beijing 100871, China

[d]Beijing Advanced Innovation Center for Materials Genome Engineering, Institute of Solid State Chemistry, University of Science and Technology Beijing, Beijing 100083, China

[e]School of Science, Hainan University, Haikou 570228, China

Email: xing@ustb.edu.cn; limanrong@hainanu.edu.cn

[#] These authors contributed equally.

SUPPLEMENTARY TEXT

V-based Chemical Pressure Calibration

Although the local structure changes can be very anisotropic, the unit cell volume (V) undergoes consistent contraction under either incremental P_{phy} or P_{chem}. Here, we employ V to calibrate the P_{chem} as a reference for the following studies. The V-based criterion has developed a connection between P_{phy} and V-scaling P_{chem}, and quantified the P_{chem} in unit-cell dimension. The P-V relation is described by the second-order Birch-Murnaghan equation of state (Equation (Eq. [S1]) has been proven to be effective [1,2].

$$P(V) = \frac{3B_0}{2}\left[\left(\frac{V_0}{V}\right)^{\frac{7}{3}} - \left(\frac{V_0}{V}\right)^{\frac{5}{3}}\right]\left\{1 + \frac{3}{4}(B' - 4)\left[\left(\frac{V_0}{V}\right)^{\frac{2}{3}} - 1\right]\right\} \quad \text{[S1]}$$

Experimentally, P_{phy} can be monitored by observing the spectral shift of the sharp fluorescent R_1 Ruby line in DAC [3], and P_{phy} -related V/Z (Z = 4) can be derived from the refinements of *in situ* high-pressure synchrotron PXD data of $RE_{0.98}Bi_{0.02}VO_4$. In **Figure S9a**, the P_{phy} -V/Z data were fitted by the second-order Birch-Murnaghan equation of state, where the first derivative of the bulk modulus with respect to pressure, B', is fixed to be 4 (R^2 = 0.999), yielding the zero-pressure bulk modulus B_0 of 250.6 GPa and a lattice volume at zero pressure (V_0) of 79.4 Å^3 for $Y_{0.98}Bi_{0.02}VO_4$. The Sc^{3+} substitution in $Y_{0.98-x}Sc_xBi_{0.02}VO_4$ successfully simulates the cell compression under P_{phy}. Thus, we assume that the V effect is equivalent to that of the x-dependent cell evolution (x = 0.1 - 0.9) to quantify the cell compression effect by Sc^{3+}-doping. To obtain the corresponding P_{chem} values, as shown in **Table 1**, we applied the refined x-dependent V of $Y_{0.98-x}Sc_xBi_{0.02}VO_4$ into Eq. [S1] along with the

previously obtained B_0 and B' values. The P_{chem}-V/Z data and Eq. [S1] curve (blue) of the matching is shown in Figure S9b, which modeled well the relationship between P_{chem} and structure compression in the unit-cell dimension. The P_{chem} was similarly quantified in $Gd_{0.98}Bi_{0.02}VO_4$ (**Fig. S9c** and **d**), where B_0 = 156.7 GPa, B' = 4 (fixed), and V_0 = 82.3 Å^3, are applied (**Fig. S9c**), respectively. The goodness of fitting R^2 = 0.991 close to 1 indicates decent simulation reliability. The corresponding P_{chem} values of $Gd_{0.98\text{-}x}Sc_xBi_{0.02}VO_4$ and P_{chem}-V/Z data with the curve (green) of the Eq. [S1] is shown in **Table 1** and **Figure S9d**, respectively. This V-based method has been widely adopted to evaluate P_{chem} [4], however, it cannot really portray the pressure passed to local structure, since negative local pressure (expansion) can occur given a positive (contraction) P_{chem} [2]. Thus, the scaling of $P_{\text{chem-}\Delta}$ by P_{chem} is incapable, it is urgently desired to develop an experimental approach to calibrate the pressure sensed by local structure.

SUPPLEMENTARY FIGURES

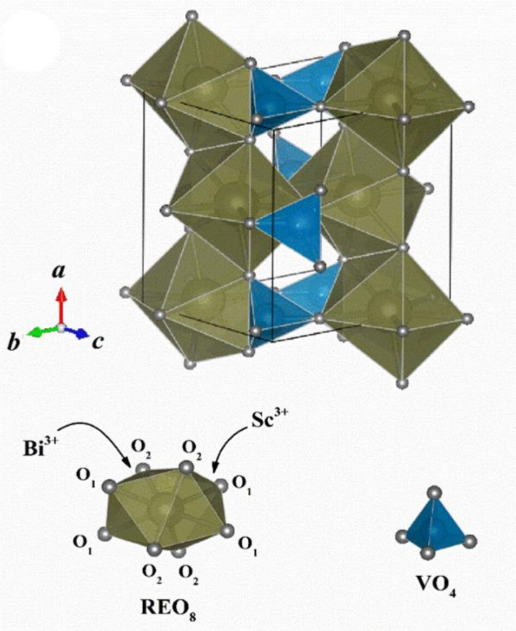

Figure S1. Crystal structure of *RE*VO_4 host and the coordination environments of cations.

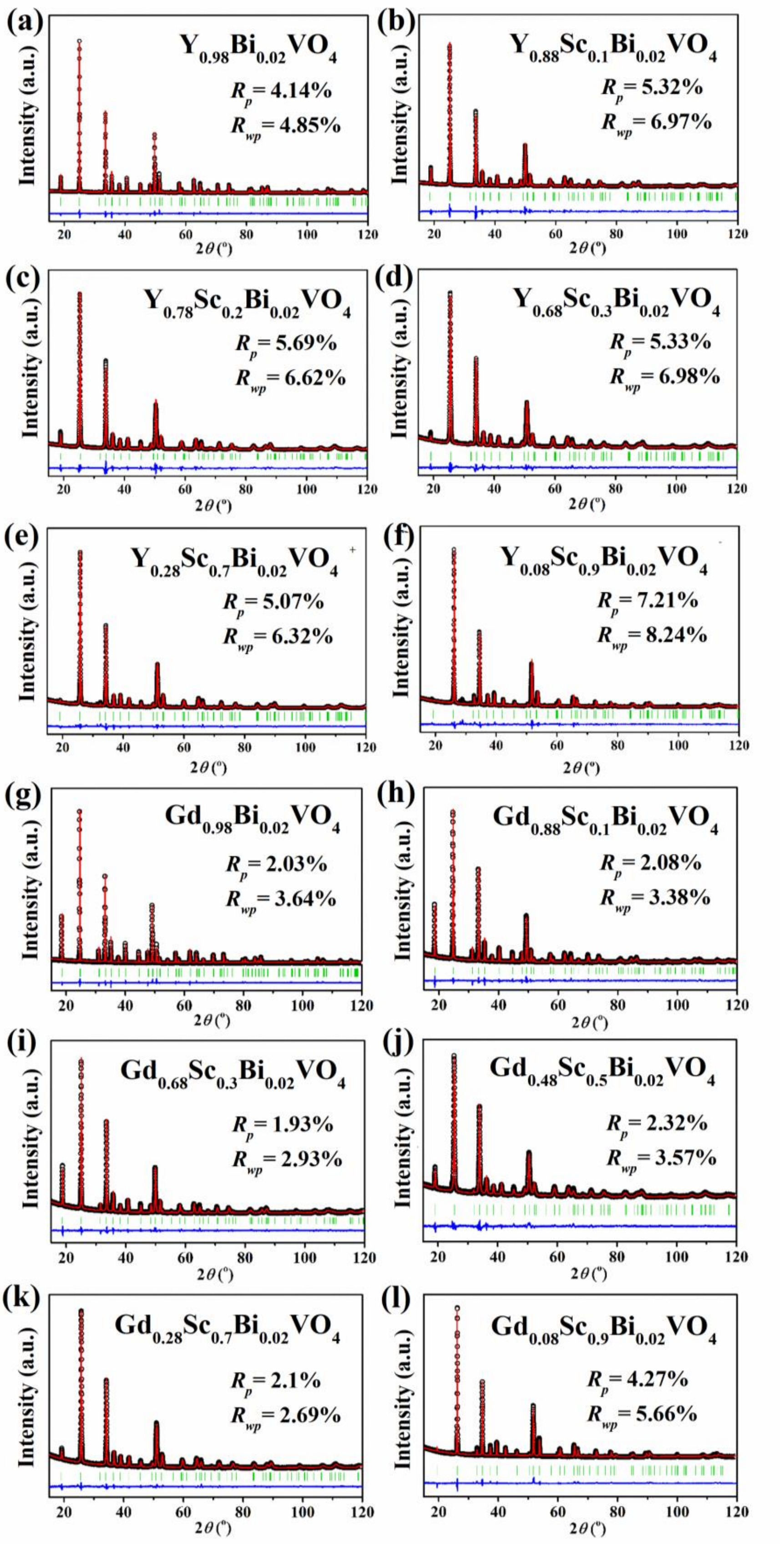

Figure S2. Rietveld refinement results of the PXD patterns of $RE_{0.98\text{-}x}Sc_xBi_{0.02}VO_4$ (RE = Y, Gd; $0 \leq x \leq 0.9$), and λ = 0.15418 nm.

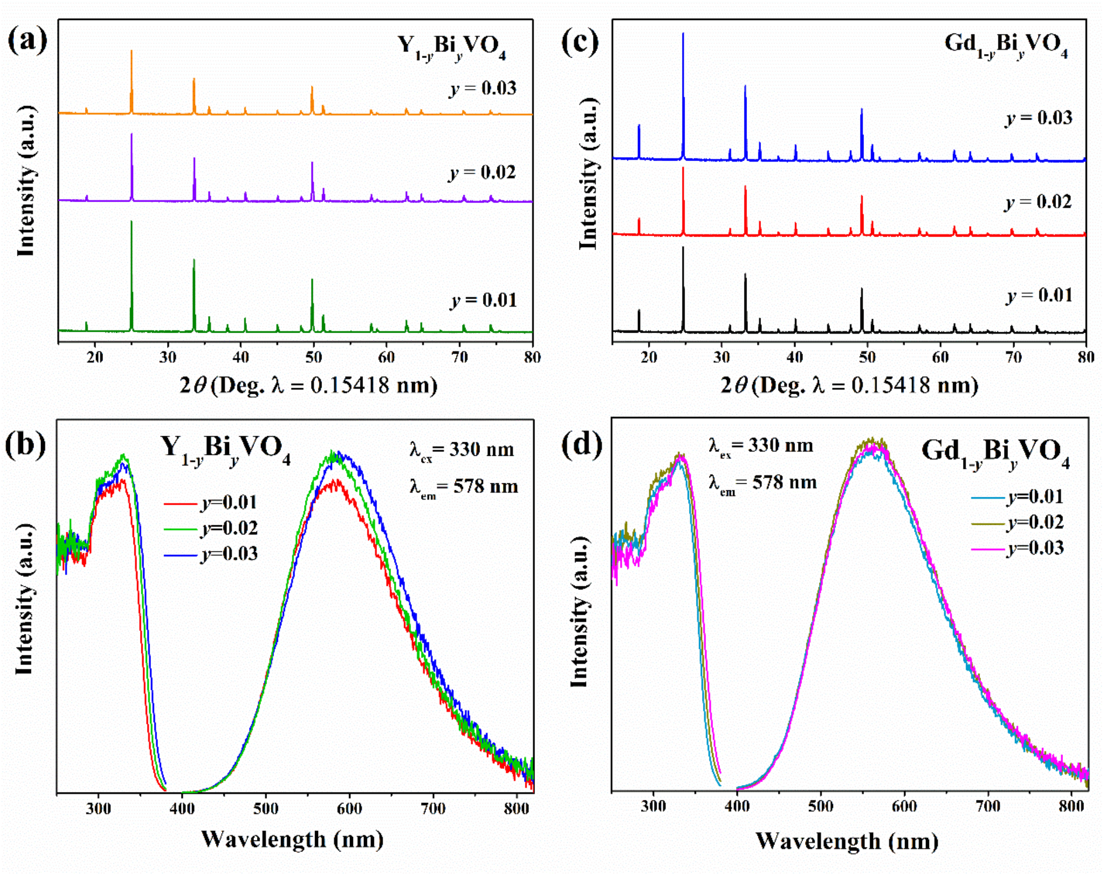

Figure S3. PXD patterns and excitation, emission spectra of (a, b) $Y_{1-y}Bi_yVO_4$, (c, d) $Gd_{1-y}Bi_yVO_4$ (y = 0.01, 0.02, 0.03).

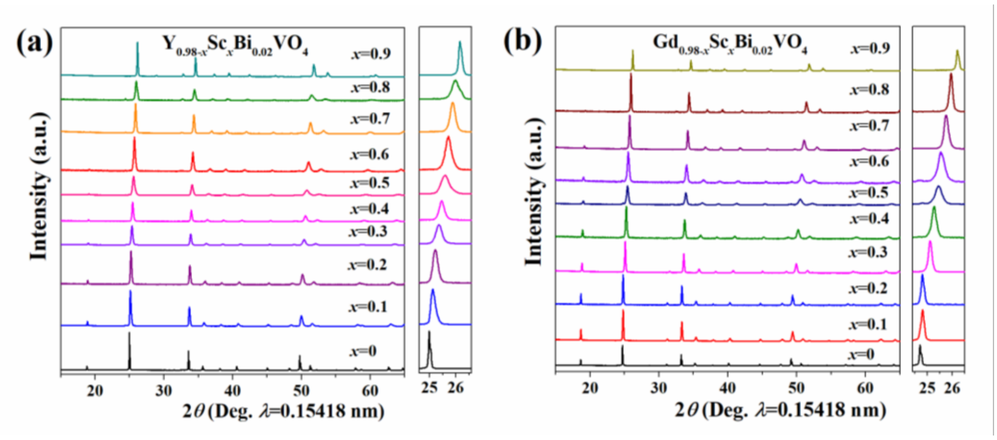

Figure S4. Concentration-dependent PXD patterns of $Y_{0.98-x}Sc_xBi_{0.02}VO_4$ (a) and $Gd_{0.98-x}Sc_xBi_{0.02}VO_4$ (b).

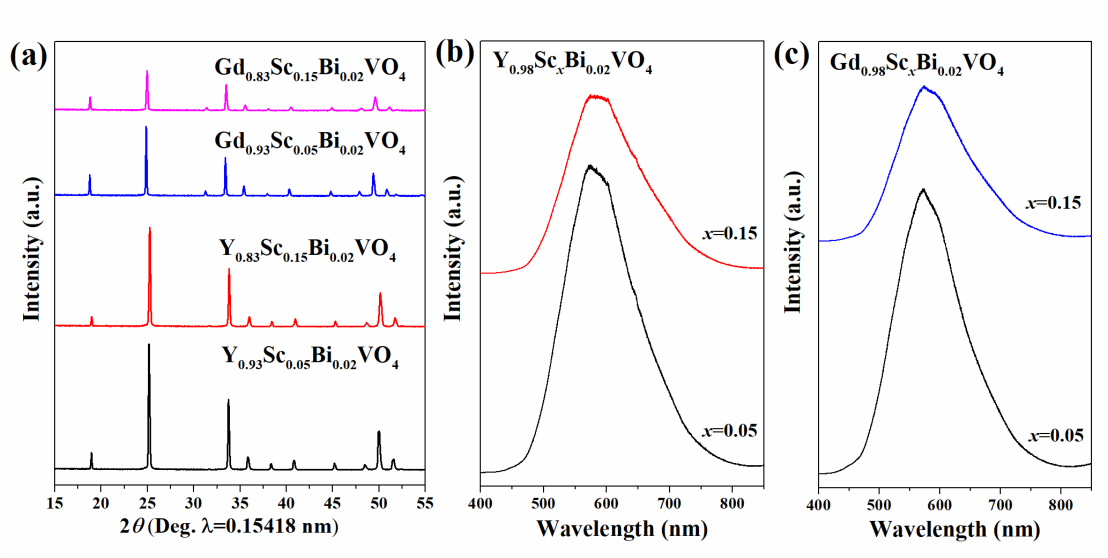

Figure S5. (a) PXD patterns and emission spectra of (b) $Y_{0.98-x}Sc_xBi_{0.02}VO_4$, (c) $Gd_{0.98-x}Sc_xBi_{0.02}VO_4$ for x = 0.05, 0.15.

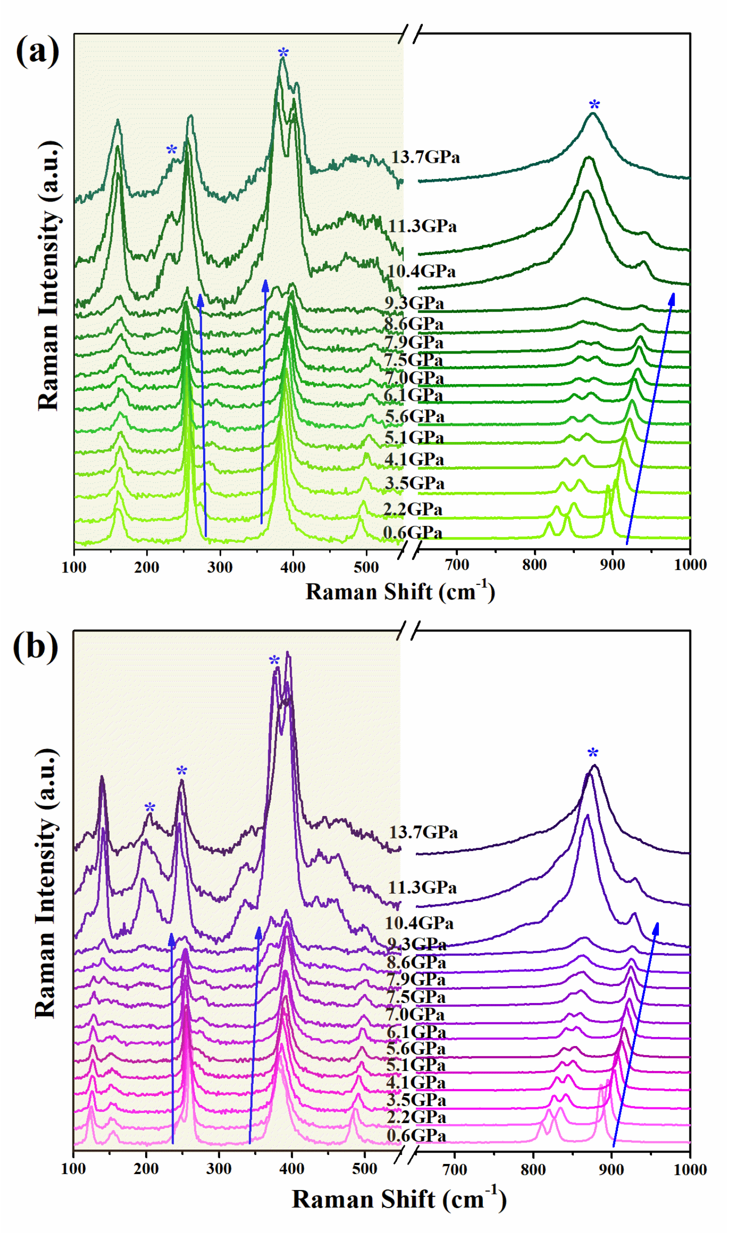

Figure S6. Pressure-dependent Raman spectra of $Y_{0.98}Bi_{0.02}VO_4$ (a) and $Gd_{0.98}Bi_{0.02}VO_4$ (b).

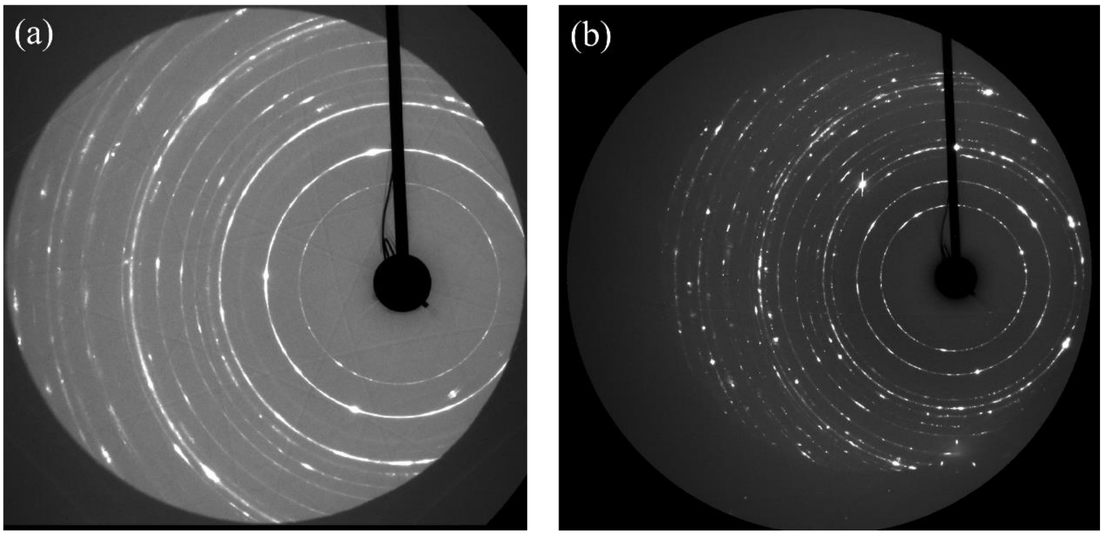

Figure S7. *In situ* high-pressure SPXD patterns of (a) $Y_{0.98}Bi_{0.02}VO_4$ at 1.3 GPa and (b) $Gd_{0.98}Bi_{0.02}VO_4$ at 1.4 GPa to show the overexposure dots.

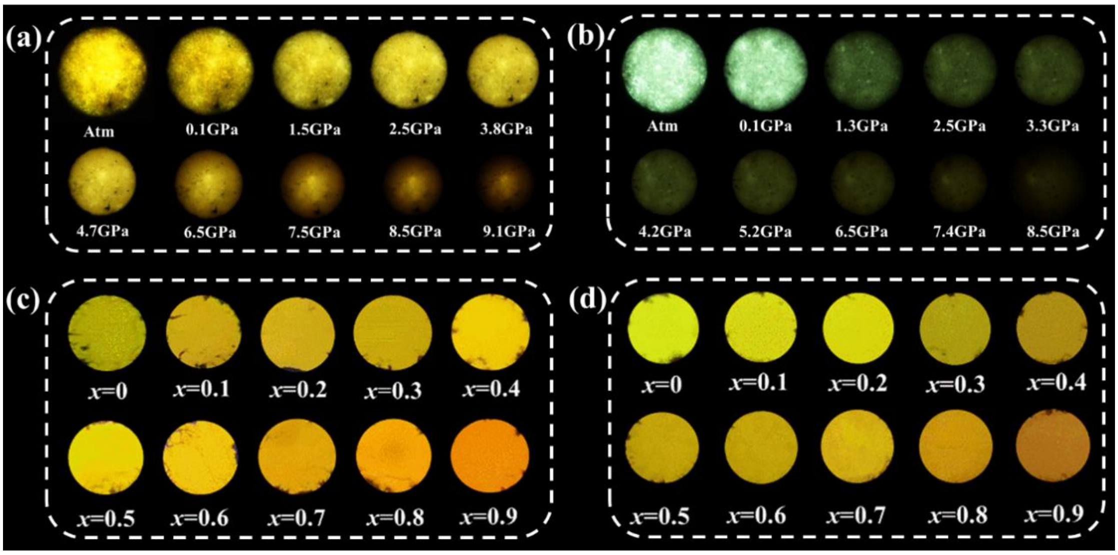

Figure S8. Pressure-dependence color evolution of $Y_{0.98}Bi_{0.02}VO_4$ (a) and $Gd_{0.98}Bi_{0.02}VO_4$ (b) (excited by 330 nm) in the microscopic field of a view (200 μm) with the camera system. Photographed color evolution of $Y_{0.98-x}Sc_xBi_{0.02}VO_4$ (c) and $Gd_{0.98-x}Sc_xBi_{0.02}VO_4$ (d) with different ion substitution concentration x in the black box with 325 nm source.

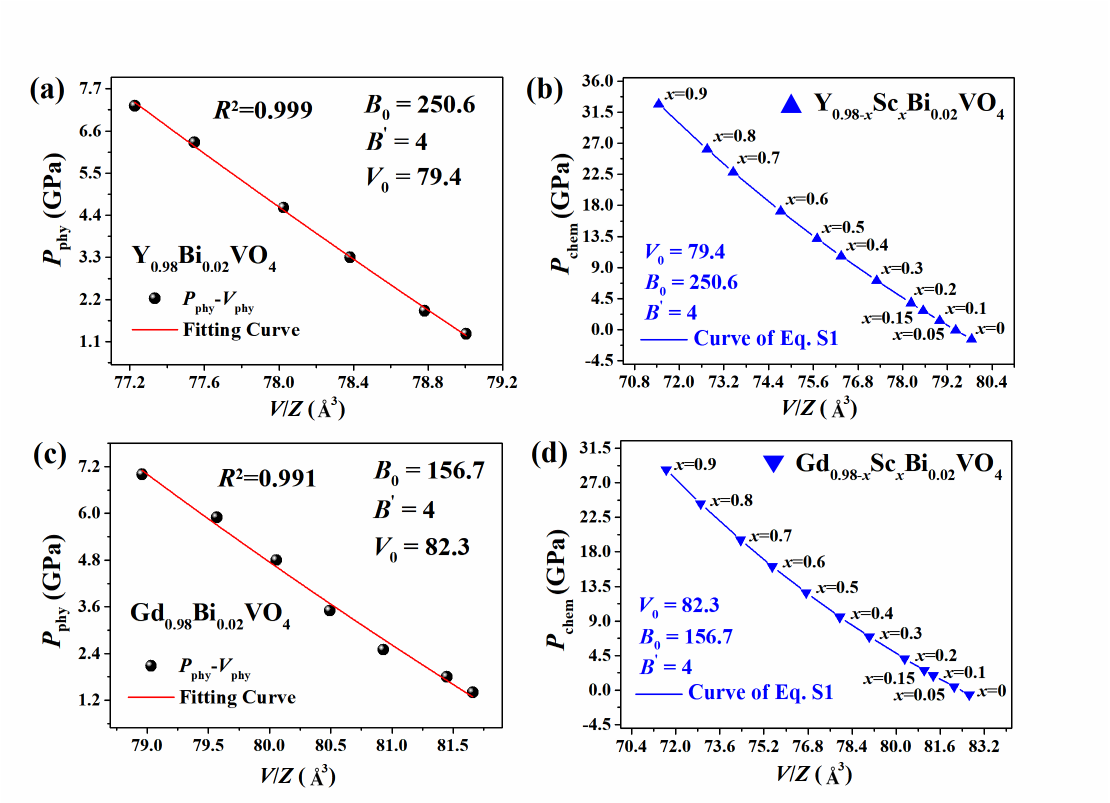

Figure S9. The V-based chemical pressure calibration for $RE_{0.98\text{-}x}Sc_xBi_{0.02}VO_4$ ($0 \leq x \leq 0.9$): Evolution of V/Z under P_{phy} of $Y_{0.98}Bi_{0.02}VO_4$ (a) and $Gd_{0.98}Bi_{0.02}VO_4$ (c) at high pressure fitted by the first-order Birch-Murnaghan equation of state. The P_{chem} versus V/Z data of $Y_{0.98\text{-}x}Sc_xBi_{0.02}VO_4$ (b) and $Gd_{0.98\text{-}x}Sc_xBi_{0.02}VO_4$ (d) with the Eq. [S1] curve.

SUPPLEMENTARY TABLES AND CAPTIONS

Table S1. Crystallographic data of (0 ≤ *x* ≤ 0.9) $Y_{0.98-x}Sc_xBi_{0.02}VO_4$ and $Gd_{0.98-x}Sc_xBi_{0.02}VO_4$ ($Y_{0.98}Bi_{0.02}VO_4$ and $Gd_{0.98}Bi_{0.02}VO_4$) refined from the room-temperature PXD (pressure dependent synchrotron radiation) data.

**$Y_{0.98-x}Sc_xBi_{0.02}VO_4$**							
Concentration(%)	*x*= 0	*x*= 0.1	*x*= 0.3	*x*= 0.5	*x*= 0.7	*x*= 0.9	
S. G.			$I4_1/amd$				
a (Å)	7.1235(1)	7.0928(6)	7.0306(10)	6.9711(13)	6.8893(10)	6.8143(5)	
c (Å)	6.2944(0)	6.2815(6)	6.2558(9)	6.2302(11)	6.1898(8)	6.1557(5)	
Z			4				
θ range			10-120°				
V (Å^3)	319.4(0)	316.0(1)	309.2(1)	302.8(1)	293.8(1)	285.8(0)	
R_{wp} (%)	4.85	6.97	6.62	6.98	6.32	8.24	
R_p (%)	4.14	5.32	5.69	5.33	5.07	7.21	
**$Y_{0.98}Bi_{0.02}VO_4$**							
Pressure (GPa)	1.3	1.9	3.3	4.6	6.3	7.3	
S. G.			$I4_1/amd$				
a (Å)	7.0948(8)	7.0866(3)	7.0713(4)	7.0565(4)	7.0377(5)	7.0265(5)	
c (Å)	6.2782(7)	6.2750(4)	6.2702(5)	6.2669(5)	6.2628(6)	6.2569(7)	
Z			4				
θ range			5-27°				
V (Å^3)	316.02(8)	315.13(4)	313.53(4)	312.05(5)	310.19(5)	308.91(5)	
R_{wp} (%)	17.05	15.44	17.79	19.24	20.6	21.11	
R_p (%)	9.26	9.31	12.17	13.42	14.98	15.44	
**$Gd_{0.98-x}Sc_xBi_{0.02}VO_4$**							
Concentration(%)	*x*= 0	*x*= 0.1	*x*= 0.3	*x*= 0.5	*x*= 0.7	*x*= 0.9	
S. G.			$I4_1/amd$				
a (Å)	7.2151(1)	7.1696(3)	7.0876(5)	7.0041(9)	6.9195(6)	6.8209(3)	
c (Å)	6.3512(1)	6.3298(3)	6.2924(4)	6.2559(8)	6.2117(5)	6.1598(3)	
Z			4				
θ range			10-120°				
V (Å^3)	330.6(0)	325.4(0)	316.1(0)	306.9(1)	297.4(1)	286.6(0)	
R_{wp} (%)	3.64	3.38	2.93	3.57	2.69	5.66	
R_p (%)	2.03	2.08	1.93	2.32	2.1	4.27	
**$Gd_{0.98}Bi_{0.02}VO_4$**							
Pressure (GPa)	1.4	1.8	2.5	3.5	4.8	5.9	7
S. G.			$I4_1/amd$				
a (Å)	7.181(1)	7.176(5)	7.159(9)	7.145(14)	7.128(10)	7.110(9)	7.091(11)
c (Å)	6.334(1)	6.327(5)	6.317(8)	6.307(12)	6.302(9)	6.297(8)	6.281(9)
Z			4				

θ range				5-28°			
V (Å^3)	326.6(1)	325.8(5)	323.7(9)	322.0(14)	320.2(10)	318.3(10)	315.8(8)
R_{wp} (%)	8.67	9.24	11.11	9.91	11.57	11.12	14.71
R_p (%)	11.2	11.67	14.27	12.93	14.28	13.27	16.96

Table S2. Atomic coordinates, occupancies, the bond lengths, and angle values of refined (*RE* = Y, Gd) $RE_{0.98\text{-}x}Sc_xBi_{0.02}VO_4$.

**$Y_{0.98\text{-}x}Sc_xBi_{0.02}VO_4$**						
***x* = 0**						
	x	***y***	***z***	***Ox.***	***occ.***	***Biso*(Å^2)**
Y	0	0.75	0.125	+3	0.98	0.4
V	0	0.25	0.375	+5	1	0.4
O	0	0.4267(1)	0.2070(1)	-2	1	0.1
Bi	0	0.75	0.125	+3	0.02	0.4
**Bond**	***d*(Å)**		**Angle(°)**		**Angle(°)**	
Y/Bi-O2	2.3600(9)×4	O-Y/Bi-O	92.7(0)	O-V-O	114.4(0)	
Y/Bi-O1	2.4400(9)×4		154.7(0)		100.0(0)	
V-O	1.6439(9)×4		71.6(0)			
			79.2(0)			
			133.7(0)			
			137.2(0)			
			62.1(0)			
***x* = 0.1**						
	x	***y***	***z***	***Ox.***	***Occ.***	***Biso*(Å^2)**
Y	0	0.75	0.125	+3	0.88	0.8(0)
V	0	0.25	0.375	+5	1	0.8(0)
O	0	0.4303(2)	0.2093(2)	-2	1	1.4(1)
Bi	0	0.75	0.125	+3	0.02	0.8(0)
Sc	0	0.75	0.125	+3	0.1	0.8(0)
**Bond**	***d*(Å)**		**Angle(°)**		**Angle(°)**	
Y/Bi-O2	2.3283(14)×4	O-Y/Bi-O	93.0(0)	O-V-O	113.5(1)	
Y/Bi-O1	2.4587(15)×4		153.7(1)		101.7(1)	
V-O	1.6491(14)×4		71.8(0)			
			78.8(0)			
			134.5(0)			
			136.8(0)			
			62.7(0)			
***x* = 0.3**						
	x	***y***	***z***	***Ox.***	***Occ.***	***Biso*(Å^2)**
Y	0	0.75	0.125	+3	0.68	1.1(0)
V	0	0.25	0.375	+5	1	1.1(0)
O	0	0.4352(2)	0.2093(3)	-2	1	2.1(1)

Bi	0	0.75	0.125	+3	0.02	1.1(0)
Sc	0	0.75	0.125	+3	0.3	1.1(0)
**Bond**	***d*(Å)**		**Angle(°)**		**Angle(°)**	
Y/Bi-O2	2.2752(16)×4	O-Y/Bi-O	93.1(0)	O-V-O	112.8(1)	
Y/Bi-O1	2.4635(17)×4		153.2(1)		103.0(1)	
V-O	1.6644(16)×4		71.5(1)			
			78.7(0)			
			136.1(1)			
			135.3(0)			
			63.8(1)			
***x* = 0.5**						
	x	***y***	***z***	***Ox.***	***Occ.***	***Biso*(Å^2)**
Y	0	0.75	0.125	+3	0.48	0.7(1)
V	0	0.25	0.375	+5	1	1.8(1)
O	0	0.4401(3)	0.2019(4)	-2	1	1.5(1)
Bi	0	0.75	0.125	+3	0.02	0.7(1)
Sc	0	0.75	0.125	+3	0.5	0.7(1)
**Bond**	***d*(Å)**		**Angle(°)**		**Angle(°)**	
Y/Bi-O2	2.2130(20)×4	O-Y/Bi-O	92.7(0)	O-V-O	113.5(1)	
Y/Bi-O1	2.4290(20)×4		155.0(1)		101.7(2)	
V-O	1.7090(20)×4		79.6(1)			
			69.4(1)			
			135.6(1)			
			66.1(0)			
			134.6(1)			
***x* = 0.7**						
	x	***y***	***z***	***Ox.***	***Occ.***	***Biso*(Å^2)**
Y	0	0.75	0.125	+3	0.28	0.5(0)
V	0	0.25	0.375	+5	1	0.5(0)
O	0	0.4502(3)	0.1954(4)	-2	1	0.5(0)
Bi	0	0.75	0.125	+3	0.02	0.5(0)
Sc	0	0.75	0.125	+3	0.7	0.5(1)
**Bond**	***d*(Å)**		**Angle(°)**		**Angle(°)**	
Y/Bi-O2	2.1112(18)×4	O-Y/Bi-O	92.4(0)	O-V-O	113.2(1)	
Y/Bi-O1	2.4150(20)×4		156.2(1)		102.3(1)	
V-O	1.7710(20)×4		67.1(1)			
			80.2(1)			
			136.7(1)			
			69.6(1)			
			132.4(1)			
***x* = 0.9**						
	x	***y***	***z***	***Ox.***	***Occ.***	***Biso*(Å^2)**
Y	0	0.75	0.125	+3	0.08	0.5(0)
V	0	0.25	0.375	+5	1	0.5(0)

O	0	0.4502(3)	0.1954(4)	-2	1	0.5(0)
Bi	0	0.75	0.125	+3	0.02	0.5(0)
Sc	0	0.75	0.125	+3	0.9	0.5(1)
**Bond**	***d*(Å)**		**Angle(°)**		**Angle(°)**	
Y/Bi-O2	2.1112(18)×4	O-Y/Bi-O	92.4(0)	O-V-O	113.2(1)	
Y/Bi-O1	2.4150(20)×4		156.2(1)		102.3(1)	
V-O	1.7710(20)×4		67.1(1)			
			80.2(1)			
			136.7(1)			
			69.6(1)			
			132.4(1)			
			**$Gd_{0.98-x}Sc_xBi_{0.02}VO_4$**			
***x* = 0**						
	x	***y***	***z***	***Ox.***	***Occ.***	***Biso*(Å^2)**
Gd	0	0.75	0.125	+3	0.98	0.5(8)
V	0	0.25	0.375	+5	1	0.5(1)
O	0	0.0725(2)	0.2134(2)	-2	1	0.5(1)
Bi	0	0.75	0.125	+3	0.02	0.5(1)
**Bond**	***d*(Å)**		**Angle(°)**		**Angle(°)**	
Gd/Bi-O2	2.3939(14)×4	O-Y/Bi-O	93.2(0)	O-V-O	113.0(1)	
Gd/Bi-O1	2.5015(13)×4		152.9(1)		102.6(1)	
V-O	1.6412(13)×4		78.4(0)			
			72.8(1)			
			134.3(0)			
			137.6(0)			
			61.6(1)			
***x* = 0.1**						
	x	***y***	***z***	***Ox.***	***Occ.***	***Biso*(Å^2)**
Gd	0	0.75	0.125	+3	0.88	0.5(0)
V	0	0.25	0.375	+5	1	0.5(0)
O	0	0.0652(2)	0.2130(3)	-2	1	0.5(1)
Bi	0	0.75	0.125	+3	0.02	0.5(0)
Sc	0	0.75	0.125	+3	0.1	0.5(0)
**Bond**	***d*(Å)**		**Angle(°)**		**Angle(°)**	
Y/Bi-O2	2.3277(16)×4	O-Y/Bi-O	93.3(0)	O-V-O	112.0(0)	
Y/Bi-O1	2.5166(16)×4		152.3(1)		104.5(1)	
V-O	1.6751(15)×4		72.1(1)			
			78.3(0)			
			135.6(0)			
			63.5(1)			
			136.3(1)			
***x* = 0.3**						
	x	***y***	***z***	***Ox.***	***Occ.***	***Biso*(Å^2)**
Gd	0	0.75	0.125	+3	0.68	0.5(0)

V	0	0.25	0.375	+5	1	0.5(0)
O	0	0.0631(2)	0.2090(2)	-2	1	0.6(1)
Bi	0	0.75	0.125	+3	0.02	0.5(0)
Sc	0	0.75	0.125	+3	0.3	0.5(0)
**Bond**	***d*(Å)**		**Angle(º)**		**Angle(º)**	
Y/Bi-O2	2.2811(13)×4	O-Y/Bi-O	93.1(0)	O-V-O	112.5(1)	
Y/Bi-O1	2.4843(13)×4		153.2(1)		103.5(1)	
V-O	1.6871(13)×4		78.7(1)			
			71.2(1)			
			135.6(0)			
			64.5(1)			
			135.7(1)			
***x* = 0.5**						
	x	***y***	***z***	***Ox.***	***Occ.***	***Biso*(Å^2)**
Gd	0	0.75	0.125	+3	0.48	0.5(0)
V	0	0.25	0.375	+5	1	0.5(1)
O	0	0.0590(2)	0.2172(3)	-2	1	1.7(1)
Bi	0	0.75	0.125	+3	0.02	0.5(0)
Sc	0	0.75	0.125	+3	0.5	0.5(0)
**Bond**	***d*(Å)**		**Angle(º)**		**Angle(º)**	
Y/Bi-O2	2.2401(16)×4	O-Y/Bi-O	93.8(0)	O-V-O	110.7(1)	
Y/Bi-O1	2.5243(18)×4		150.2(1)		107.2(1)	
V-O	1.6623(17)×4		72.9(1)			
			77.4(0)			
			136.9(1)			
			64.0(1)			
			136.0(1)			
***x* = 0.7**						
	x	***y***	***z***	***Ox.***	***Occ.***	***Biso*(Å^2)**
Gd	0	0.75	0.125	+3	0.28	0.5(0)
V	0	0.25	0.375	+5	1	0.5(1)
O	0	0.0612(1)	0.2045(2)	-2	1	1.0(0)
Bi	0	0.75	0.125	+3	0.02	0.5(0)
Sc	0	0.75	0.125	+3	0.7	0.5(0)
**Bond**	***d*(Å)**		**Angle(º)**		**Angle(º)**	
Y/Bi-O2	2.2091(10)×4	O-Y/Bi-O	92.9(0)	O-V-O	113.4(0)	
Y/Bi-O1	2.4285(11)×4		154.2(1)		102.0(1)	
V-O	1.6818(10)×4		79.1(0)			
			70.4(1)			
			135.5(0)			
			65.1(1)			
			135.3(0)			
***x* = 0.9**						
	x	***y***	***z***	***Ox.***	***Occ.***	***Biso*(Å^2)**

Gd	0	0.75	0.125	+3	0.08	0.5(3)
V	0	0.25	0.375	+5	1	0.5(1)
O	0	0.0500(2)	0.1962(3)	-2	1	0.5(1)
Bi	0	0.75	0.125	+3	0.02	0.5(6)
Sc	0	0.75	0.125	+3	0.9	0.5(5)
**Bond**	***d*(Å)**		**Angle(^{o})**		**Angle(^{o})**	
Y/Bi-O2	2.0926(15)×4	O-Y/Bi-O	92.5(0)	O-V-O	113.2(1)	
Y/Bi-O1	2.4035(17)×4		155.8(1)		102.2(1)	
V-O	1.7534(16)×4		80.1(0)			
			67.5(1)			
			136.7(1)			
			69.2(1)			
			132.7(1)			

SUPPLEMENTARY REFERENCES

1 Birch F. Finite Elastic Strain of Cubic Crystals. *Phys Rev* 1947; **71**: 809-24.

2 Zhou X, Zhao M H and Yang J. Chemical Pressure Enlarged Camouflage Color Zone in Mn(IV)-Activated Yellow-Green Pigments. *Mater Today Chem* 2022; **25**: 100902.

3 Mao H K, Xu J and Bell P M. Calibration of the Ruby Pressure Gauge to 800 Kbar under Quasi-Hydrostatic Conditions. *J Geophys Res (USA)* 1986; **91**: 4673-6.

4 Das P, Kanchanavatee N, Helton J S, Huang K, Baumbach R E, Bauer E D, White B D, Burnett V W, Maple M B, Lynn J W and Janoschek M. Chemical Pressure Tuning of URu_2Si_2 via Isoelectronic Substitution of Ru with Fe. *Phys Rev B* 2015; **91**: 085122.
